# Analogues of Natural Chalcones as Efficient Inhibitors of AKR1C3

**DOI:** 10.3390/metabo12020099

**Published:** 2022-01-21

**Authors:** Gabriele Möller, Veronika Temml, Antonio Cala Peralta, Océane Gruet, Pascal Richomme, Denis Séraphin, Guillaume Viault, Luisa Kraus, Petra Huber-Cantonati, Elisabeth Schopfhauser, Johanna Pachmayr, Janina Tokarz, Daniela Schuster, Jean-Jacques Helesbeux, Kenneth Allen Dyar

**Affiliations:** 1 Institute for Diabetes and Cancer, Helmholtz Center Munich, German Research Center for Environmental Health, 85764 Neuherberg, Germany; janina.tokarz@helmholtz-muenchen.de (J.T.); kenneth.dyar@helmholtz-muenchen.de (K.A.D.); 2 Department of Pharmaceutical and Medicinal Chemistry, Institute of Pharmacy, Paracelsus Medical University Salzburg, 5020 Salzburg, Austria; veronika.temml@pmu.ac.at (V.T.); Elisabeth.Schopfhauser@gmx.at (E.S.); daniela.schuster@pmu.ac.at (D.S.); 3 University of Angers, SONAS, SFR QUASAV, F-49000 Angers, France; antonio.calaperalta@univ-angers.fr (A.C.P.); o.gruethuyghe@gmail.com (O.G.); pascal.richomme@univ-angers.fr (P.R.); denis.seraphin@univ-angers.fr (D.S.); guillaume.viault@univ-angers.fr (G.V.); jean-jacques.helesbeux@univ-angers.fr (J.-J.H.); 4 Institute of Pharmacy, Pharmaceutical Biology and Clinical Pharmacy, Paracelsus Medical University Salzburg, 5020 Salzburg, Austria; luisa.kraus@pmu.ac.at (L.K.); petra.cantonati@pmu.ac.at (P.H.-C.); johanna.pachmayr@pmu.ac.at (J.P.)

**Keywords:** chalcone, aldo-keto reductase, cancer, AKR1C3, 17β-hydroxysteroid dehydrogenase, 3α-hydroxysteroid dehydrogenase, structure-activity relationship

## Abstract

Naturally occurring substances are valuable resources for drug development. In this respect, chalcones are known to be antiproliferative agents against prostate cancer cell lines through various mechanisms or targets. Based on the literature and preliminary results, we aimed to study and optimise the efficiency of a series of chalcones to inhibit androgen-converting AKR1C3, known to promote prostate cancer. A total of 12 chalcones with different substitution patterns were synthesised. Structure–activity relationships associated with these modifications on AKR1C3 inhibition were analysed by performing enzymatic assays and docking simulations. In addition, the selectivity and cytotoxicity of the compounds were assessed. In enzymatic assays, C-6′ hydroxylated derivatives were more active than C-6′ methoxylated derivatives. In contrast, C-4 methylation increased activity over C-4 hydroxylation. Docking results supported these findings with the most active compounds fitting nicely in the binding site and exhibiting strong interactions with key amino acid residues. The most effective inhibitors were not cytotoxic for HEK293T cells and selective for 17β-hydroxysteroid dehydrogenases not primarily involved in steroid hormone metabolism. Nevertheless, they inhibited several enzymes of the steroid metabolism pathways. Favourable substitutions that enhanced AKR1C3 inhibition of chalcones were identified. This study paves the way to further develop compounds from this series or related flavonoids with improved inhibitory activity against AKR1C3.

## 1. Introduction

Aldo–keto reductase 1C3 (AKR1C3), also called 17β-hydroxysteroid dehydrogenase (17β-HSD) type 5, belongs to the large superfamily of aldo–keto reductases which is divided into 15 subfamilies. In humans, AKR1C3, together with AKR1C1, AKR1C2, and AKR1C4, constitutes the AKR1C family of hydroxysteroid dehydrogenases (https://hosting.med.upenn.edu/akr/, accessed on 10 December 2021). AKR1C3 is a monomeric enzyme of 37 kDa, displaying a TIM-barrel structure with a characteristic (αβ)8-barrel motif [1] typical for all AKRs. The cytosolic enzyme is expressed in many steroidogenic tissues and steroid hormone target tissues [2,3], including testis, prostate, uterus, ovary, and breast [4,5,6].

AKR1C3 is a NAD(P)H-dependent enzyme catalysing the reduction of endogenous substrates bearing aldehyde and ketone functions into their hydroxyl counterparts that can then undergo further conjugation reactions such as sulphation and glucuronidation [3].

AKR1C3 is a key enzyme in steroid hormone metabolism [7]. In its function as 3α- and 17β-hydroxysteroid dehydrogenase, this enzyme catalyses the conversion of androgens and oestrogens and is thus involved in pre-receptor regulation of the androgen receptor (AR) and oestrogen receptor (ER) signalling pathways [3]. The most important role in androgen metabolism is the generation of the two AR ligands—namely, testosterone and dihydrotestosterone (DHT). As testosterone is a substrate for aromatase-catalysed oestradiol formation, AKR1C3 also supplies the oestrogen biosynthesis pathway. In addition, AKR1C3 itself can convert estrone to oestradiol.

The enzyme also has 20α-hydroxysteroid dehydrogenase activity [8], catalysing the reduction of progesterone to 20α-hydroxyprogesterone. This activity influences the progesterone receptor (PR) pathway as well as steroid synthesis during which progesterone is an upstream intermediate in the production of other endogenous steroids such as sex steroids and glucocorticoids. The more important enzyme in progesterone breakdown is, however, AKR1C1 [9]. AKR1C3 can contribute to the supply of steroids in an endocrine manner, as testosterone was found to be synthesised by AKR1C3 in the adrenal cortex and released into the circulation [10]. However, the enzyme acts mostly in an intracrine manner, catalysing the conversion of metabolites locally within the peripheral tissues in which the metabolites are acting [1,11,12]. In addition to its enzymatic activity, AKR1C3 has been shown to function as a co-activator of the androgen receptor [13].

Moreover, AKR1C3 also plays a role in prostaglandin (PG) metabolism and is known as PGF_2_ synthase [14]. The enzyme catalyses the reduction of PGH_2_ to PGF_2α_ and PGD_2_ to 9α-11β-PGF_2α_ [14,15]. Both PGF_2α_ metabolites are able to bind to the prostaglandin receptor, inducing proliferative and inflammatory processes and thereby preventing differentiation and apoptosis [16,17].

As regulators of hormone signalling, steroid-metabolising enzymes are important in several biological processes, e.g., sexual development, reproduction, and maintenance of energy level or response to stress, and changes in their enzymatic activities can lead to serious diseases. Indeed, AKR1C3 is associated with several diseases such as prostate cancer [18], breast cancer [7], endometrial cancer [17], cervical cancer [17], endometriosis [19], non-small cell lung cancer [20], colorectal cancer [21], oropharyngeal tumour [22], leukaemia [23], gastric cancer [24], etc. [25]. Overexpression of AKR1C3 is usually observed in connection with the diseases, with the exception of gastric cancer, for which downregulation of mRNA and protein levels were reported [24]. AKR1C3 overexpression can lead to an increase in active steroids and PGF_2α_ levels in diseased tissues, thereby promoting proliferation processes. On the other hand, with its ability to reduce and deactivate drugs that carry carbonyl groups, AKR1C3 contributes to the exacerbation of disease progression. The process of drug inactivation by carbonyl reductases is part of the mechanisms of drug resistance and is a major threat to cancer therapy efficacy [6]. For example, antitumoral anthracyclines such as doxorubicin, oracin, daunorubicin, and idarubicin are metabolised into their inactive corresponding hydroxy analogues [26,27]. Since AKR1C3 is overexpressed in hormone-dependent cancers, the enzyme may contribute to the failure of treatments based on carbonyl-containing drugs. Therefore, the inhibition of reducing enzymes such as AKR1C3 represents an engaging pharmacological strategy to overcome cancer drug resistance and restore the antineoplastic activity of the drugs [28].

Many steroidal or non-steroidal AKR1C3 inhibitors have already been developed and characterised [17,25,29]. Most were developed to target AKR1C3 only, but some are both AKR1C3 inhibitors and AR antagonists [13,30,31]. Since only a few of the inhibitors have entered clinical trials, and none has made it to the application stage [25], there is still a demand for new effective AKR1C3-inhibiting compounds.

Among the various strategies used to identify promising active scaffolds or derivatives, exploring the therapeutic potential of natural products has led to major contributions and breakthroughs, especially in the field of cancer therapies. Since 2012, Newman and Cragg have deeply highlighted the importance of natural products and all the structurally related analogues in the drug discovery processes [32].

Various natural polyphenols, such as flavonoids or alkaloids, have shown significant inhibitory activities against AKR1C enzymes [33,34,35]. Chalcones, as natural secondary metabolites that belong to the class of flavonoids, are largely distributed in the plant kingdom. They have been extensively studied due to their broad range of pharmacological activities. Among them, antitumoral activities have been reported against different cancer cell lines [36,37]. Isoliquiritigenin, a tetraphenolic chalcone from liquorice, inhibited the growth of human prostate cancer cells lines DU145 and LNCaP with an IC_50_ of 11 µM and 13 µM, respectively [38]. Moreover, various biological targets have been identified to support such cytotoxicity [39,40]. Cinnamic acid derivatives, known as biosynthetic precursors of chalcones and other related flavonoids, inhibited AKR1C3, with IC_50_ generally ranging from 2 to 50 µM, except baccharin which showed a higher potency, with an IC_50_ of 100 nM [41,42]. Moreover, a library of 42 α-arylcinnamic acids was screened against the same enzymatic target. Nine exhibited an IC_50_ between 5 µM and 20 µM, and the compounds with the highest selectivity over other AKR1C enzymes had an IC_50_ of around 13.5 µM [43]. IC_50_ values from 300 nM to 50 µM were observed in studies analysing the effect of dietary flavonoids on AKR1C3 [44,45]. In a further study on 19 isoquinoline alkaloids, the most effective compounds showed AKR1C3 inhibition, with IC_50_ values of 7.7 µM and 29 µM [46]. In another study, a series of 11 chalcones were investigated for their potential to inhibit 17β-HSD activity in human microsomes [47]. Only three showed an IC_50_ below 50 µM. In terms of structural considerations, in this former study, five chalcones were either mono- or di-hydroxylated, and three showed a backbone bearing a phloroglucinol moiety.

Our current research aimed to further explore and clarify the importance of the substitution pattern on the inhibitory activity against AKR1C3 for a set of 12 synthetic chalcones (**18**–**29**), along with isoliquiritigenin (**16**) and butein (**17**) obtained from commercial sources. Our goal was to better understand the AKR1C3–chalcone interaction and to develop an efficient chalcone-based AKR1C3 inhibitor.

## 2. Results

### 2.1. Synthesis of Chalcones

**Figure 1:**
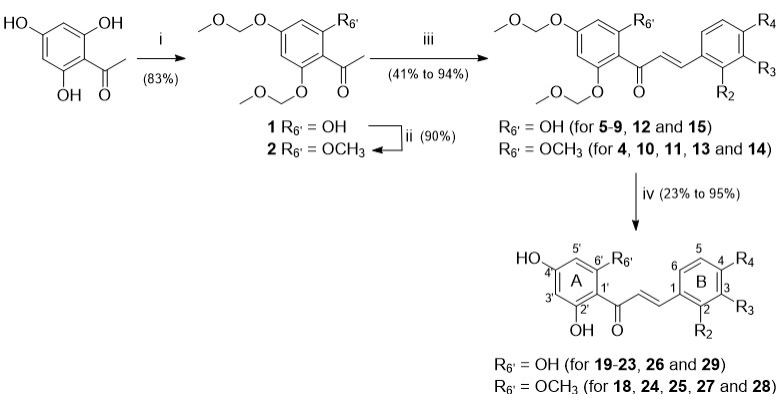
Three-step synthetic pathway to access chalcones **18–29**—reagents, conditions, and yields (in %): (**i**) MOMBr, *N*,*N*-diisopropylethylamine, THF, RT, 2 h; (**ii**) MeI, NaH, DMF, RT, 1 h; (**iii**) method A: KOH, EtOH, RT, 48 h; method B: NaOH, dioxane:water (1:1), RT, 24 h; (**iv**) method C: HCl, MeOH, RT, 40 h; method D: PTSA.H_2_O, DCM:EtOH (1:7), 60 °C, 1 h.

Chalcones **4**–**15** were synthesised using a classical approach involving a Claisen–Schmidt condensation of MOM-protected acetophenones and benzaldehydes appropriately substituted in a basic medium (Figure 1). Deprotection of MOM-chalcones was achieved in the presence of hydrochloric acid. In some instances, this led to complex mixtures, with the expected chalcones as minor products. To circumvent this drawback, removal of the protecting groups was achieved in the presence of *para*-toluenesulphonic acid [48,49].

Among chalcones **4**–**15** and **18**–**29**, the synthesis of derivatives **6**, **11**, **20**, and **25** is described here for the first time. All the other chalcones have been previously reported [48,50,51,52,53,54,55,56,57]. Chalcone **18** has also been isolated from natural sources in the frame of previous phytochemical studies [31].

The current study focused on chalcones whose substitution pattern has been modified at the C-6′ position (R_6′_ = H for isoliquiritigenin (**16**) and butein (**17**); R_6′_ = OH for **19**–**23**, **26**, and **29**; R_6′_ = OCH_3_ for **18**, **24**, **25**, **27**, and **28**) in combination with hydrophilic or lipophilic substituents (R_2_, R_3_, R_4_ = H, OH, OCH_3_, CH_3_, Cl, or F) at C-2, C-3, and/or C-4 positions (Figure 1, Table 1; Supplementary Figure S1, Supplementary Tables S1 and S2).

### 2.2. Bioactivity of Chalcones with AKR1C3 and Related Targets

#### 2.2.1. Inhibition of the Enzymatic Activity of AKR1C3

The inhibitory potency of chalcones on the catalytic activity of AKR1C3 was determined through enzymatic assays, with the measurement of the conversion of ∆4-androstene-3,17-dione to testosterone at 10 µM compound concentration. These assays shed light on a clear dependence between structural features and enzyme inhibition, with inhibition values ranging from 20.3% to 90.9% (Table 1).

**Figure 2:**
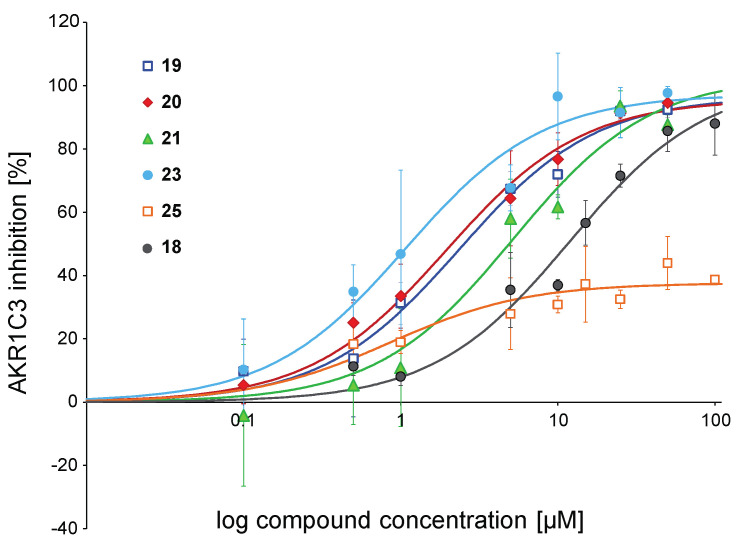
IC_50_ curves for selected chalcones. Shown is the inhibition of the ∆4-androstene-3,17-dione reduction catalysed by AKR1C3 at increasing compound concentrations. Data were fitted to the one-site ligand binding equation of the SigmaPlot Kinetics module. Symbols for compounds are shown in the plot. Experiments were performed in triplicates, and mean values ± SD are shown.

To further analyse structure-activity relationships, IC_50_ values were measured for chalcones able to inhibit AKR1C3 activity more than 75% at 10 µM. Thus, compounds **19**, **20**, **21**, and **23** were selected, along with the weak inhibitor **25** and chalcone **18** (MF-11 in [31]) which was the hit compound of the structure optimisation strategy developed in the current study. The results are presented in Table 1 and Figure 2. Chalcone **23** (IC_50_ = 1.08 µM) was the most active derivative against AKR1C3, followed by analogues **19** and **20**, with IC_50_ values in the range of 2 µM. Chalcone **21** was significantly less active than the previous three derivatives with an IC_50_ of 5.18 µM. The weaker inhibition potency of chalcone **18** was reflected in the higher IC_50_ of 11.91 µM. Compound **25** ran into saturation already at low concentrations without reaching the saturation level of 100%, so a determination of the IC_50_ value was not performed.

#### 2.2.2. Selectivity of Selected AKR1C3-Inhibiting Chalcones

**Figure 3:**
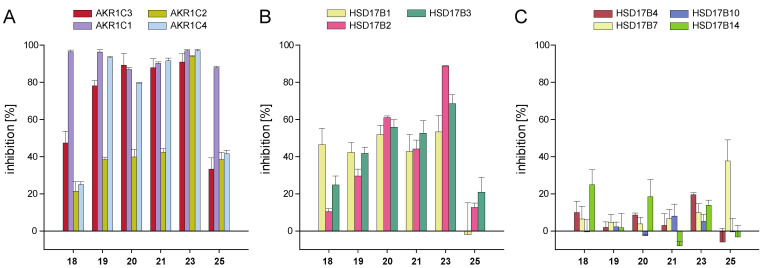
Inhibition of enzymes of the AKR1C and 17β-HSD families by selected chalcones: (**A**) inhibition of the enzymes AKR1C1, AKR1C2, and AKR1C4 in comparison with AKR1C3; (**B**) inhibition of SDR enzymes HSD17B1, HSD17B2, and HSD17B3 primarily involved in steroid hormone metabolism; (**C**) inhibition of SDR enzymes not primarily involved in steroid hormone metabolism. Experiments were performed in triplicates, and mean values ± SD are shown.

The influence of the chalcone derivatives on the activity of other human steroid hormone-metabolising enzymes was investigated in order to determine the selectivity of the compounds. To this end, the three other members of the AKR1C family were examined, along with several members of the 17β-HSD family, i.e., enzymes capable of catalysing the conversion of the same or similar substrates as AKR1C3. Screening at 10 µM compound concentration included the four strong AKR1C3 inhibitors **19**, **20**, **21**, and **23**, alongside weak inhibitors **18** and **25**. The results are shown in Figure 3 and Supplementary Tables S3–S5.

The best AKR1C3 inhibitor of our series, chalcone **23**, showed no selectivity with respect to the enzymes of the ARK1C family. This derivative also significantly inhibited HSD17B2 (88.8% at 10 µM) and to a lower extent HSD17B1 and HSD17B3, with 53.3% and 68.6% inhibition, respectively. Chalcones **19**, **20**, **21**, **18**, and **25** were weak inhibitors of AKR1C2, as they inhibited not more than 42.3% of the enzyme’s activity at 10 µM (Figure 3A). Nevertheless, **19**, **20**, and **21** were very potent inhibitors of the other three AKR1C1, AKR1C3, and AKR1C4 enzymes (>75% inhibition at 10 µM). Among the 11 enzymes considered in the current screening, AKR1C1 was the only target for **18** and **25,** with inhibition values of 96.5% and 87.9%, respectively, at 10 µM concentration (Figure 3A). All other enzymes were inhibited less than 50% by these two chalcones (Figure 3A–C). Chalcones **18**, **19**, **20,** and **21** only weakly inhibited the SDR enzymes HSD17B1, HSD17B2, and HSD17B3 (Figure 3B). All six tested compounds were nearly ineffective inhibitors of SDR enzymes HSD17B4, HSD17B7, HSD17B10, and HSD17B14 (Figure 3C).

#### 2.2.3. Cytotoxicity of Selected AKR1C3-Inhibiting Chalcones

To determine the cytotoxic effects of the selected AKR1C3-inhibiting chalcones, the viability of HEK293T cells at compound concentrations from 1 µM to 100 µM was examined. Chemotherapeutic agents cisplatin and etoposide which are conventionally used in cancer treatment were included as positive controls.

**Figure 4:**
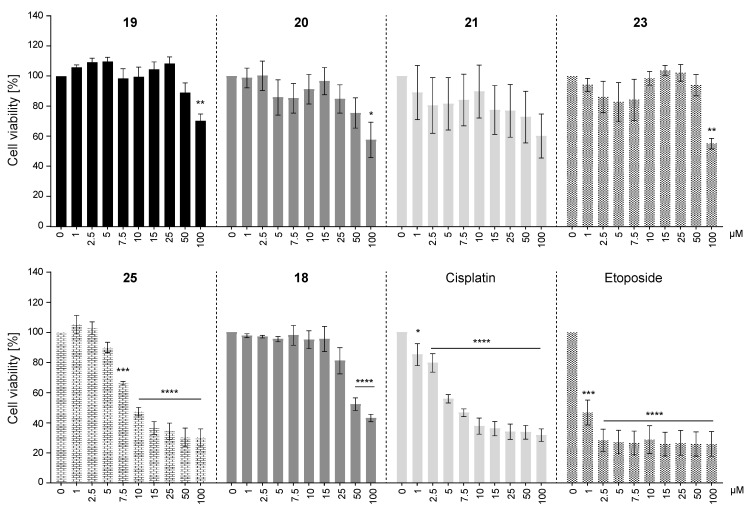
Cytotoxicity of selected chalcones on HEK293T cells. Images display cell viability of HEK293T cells that were treated with 0, 1, 2.5, 5, 7.5, 10, 15, 25, 50, or 100 µM of compounds **19**, **20**, **21**, **23**, **25**, **18**, cisplatin, or etoposide for 72 h. Cell viability at 0 µM compound concentration (vehicle) was set to 100% viability. Mean values ± SEM of three or four independent experiments are shown. One-way ANOVA (Dunnett’s test) was performed for statistical analysis, * *p* < 0.05, ** *p* < 0.005, *** *p* < 0.0002, **** *p* < 0.0001, compared with the corresponding controls (vehicle treated).

Five of the six chalcones we studied in this assay affected cell viability. Chalcone **21** was an exception, as it did not show any significant dose-response cytotoxicity (Figure 4). Viability was significantly decreased only at the highest compound concentrations of 50 µM or 100 µM for chalcones **19**, **20**, **23**, and **18**. Interestingly, **25** started to show strong and significant cytotoxic effects against HEK293T cells already at a concentration of 7.5 µM.

### 2.3. Docking and SAR Analysis for Selected AKR1C3-Inhibiting Chalcones

Docking simulations were carried out in order to explain the results from the biological evaluation of AKR1C3 activity and to characterise the structure-activity relationships between the enzyme’s binding site and the analysed chalcones. As a template for AKR1C3, we used the crystal structure in which the inhibitor 3-phenoxybenzoic acid was co-crystallised (PDB Code 3UWE) but removed before chalcone docking. The resulting docking poses for chalcones were evaluated by ranking the docking scores (Table 1) and by inspecting the positioning of the compounds within the binding pocket.

**Figure 5:**
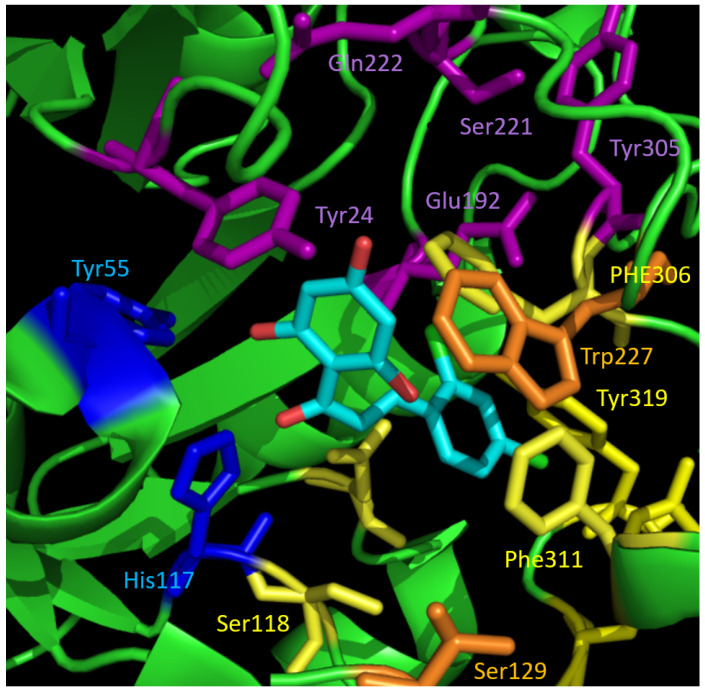
Binding pocket of AKR1C3 with the docking pose of active compound **23** (cyan). The interaction partners of the catalytic tetrad are shown in blue. The residues forming the three different subpockets SP1–3 are highlighted in different colours: SP1—yellow, SP2—orange, SP3—violet. The chalcone scaffold binds to catalytic amino acids and SP1.

**Figure 6:**
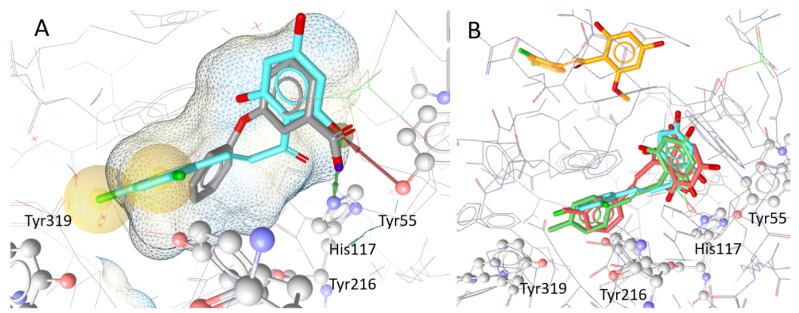
Localisation and orientation of chalcones to AKR1C3 in docking simulations: (**A**) docking pose of the protein (PDB Code: 3UWE) with its co-crystallised ligand 3-phenoxybenzoic acid (gray; IC_50_ = 0.68 µM) and compound **23** (cyan), the compound with the highest inhibitory effect. The hydrophobic interactions between compound **23** and Tyr319 and Tyr216 are highlighted in yellow. The red and green arrows indicate hydrogen bonds between the hydroxy group of ring B of **23** and Tyr55 and His117, respectively; (**B**) shows the docking poses of the highly inhibiting chalcones **23** (cyan), **20** (red), and **21** (green) in comparison with weak inhibitor **25** (orange), which was placed outside the binding pocket in simulations.

The binding site of AKR1C3 has been described as consisting of a main binding point at amino acids Tyr55 and His117 which are part of the catalytic tetrad (Tyr55, Asp50, Lys84, and His117) [9]. Moreover, three subpockets (SP1–3) exist that can be filled by ligands. Subpocket SP1 is defined by amino acids Ser118, Asn167, Phe306, Phe311, and Tyr319. Subpocket SP2 refers to Ser129 and Trp227, and the subpocket SP3 contains Tyr24, Glu192, Ser217, Ser221, Gln222, and Tyr305 [16]. Here, the investigated chalcone scaffold was shown to interact with the main binding site residues Tyr55 and His117 by forming hydrogen bonds with the hydroxy groups of ring A. Ring B filled SP1, depending on form and size (Figure 5 and Figure 6A). The compound that formed hydrophobic contacts with Tyr319 and Tyr216 in the simulation was found to be the most active compound from the dataset (Table 1). Most prominently, a high docking score, combined with a hydrophobic contact to Tyr319, correlated with a high inhibition of AKR1C3 (Table 1). Compounds with a docking score below 50 were positioned outside the binding pocket and showed only weak enzyme inhibition.

## 3. Discussion

In the course of this study, we aimed to find new effective inhibitors for the human enzyme AKR1C3. We focused on chalcones as the central core and tried to identify key substitution patterns that can enhance inhibition of AKR1C3.

Chalcone **18**, isolated from the plant *Melodorum fruticosum*, was previously identified as a mild inhibitor of AKR1C3 (MF-11; [31]) and considered here as a starting core to develop structural optimisation. While **18** exhibited 47.7% inhibition at 10 µM (with an IC_50_ of about 12 µM), two other natural phenolic chalcones, isoliquiritigenin (**16**) and butein (**17**), also included in the study, were less active (31.1% and 37.2% inhibition at 10 µM, respectively). From a structural perspective, several changes occurred in the substitution pattern of these three derivatives. First, in order to explore the importance of the methoxy substituents at the C-6′ and C-4 positions of ring A and ring B, respectively, chalcones **19** (R_4_ = OCH_3_, R_6′_ = OH; Table 1) and **24** (R_4_ = OH, R_6′_ = OCH_3_) were prepared as phenolic analogues of **18** (R_4_ = R_6′_ = OCH_3_). This led to a loss of the inhibitory potential for **24** (24.6% inhibition at 10 µM) but a significant gain for **19** with 78.1% inhibition at 10 µM. Meanwhile, pentaphenolic chalcone **29** was as active as **18** at the same concentration.

A series of derivatives with lipophilic substituents on the C-4 position were prepared and could be divided into subgroups depending on the nature of the C-6′ substituent, which was either a phenol function (**20–22**, **26**) or a methoxy group (**27** and **28**). The latter analogues of **18** bearing electron-rich substituents in C-4 position (R_4_ = Cl for **27** or R_4_ = F for **28**) were both inactive at 10 µM (26.1% and 20.3% inhibition, respectively). Corresponding compounds **22** (R_4_ = Cl) and **26** (R_4_ = F), with a phenolic function at the C-6′ position, were threefold more potent, with 70.8% and 68.5% inhibition of AKR1C3 activity at 10 µM. Two other chalcones bearing a methyl group on ring B either at the C-3 or the C-4 position, chalcones **20** and **21**, were prepared and evaluated. They both exhibited a higher inhibitory potential at 10 µM than all the chalcones mentioned before, with 89.2% and 87.9% inhibition.

As the AKR1C3-binding site can accommodate larger inhibitors [58], 2,4-dichloro ring-B analogues **23** and **25** were prepared and evaluated. Chalcone **23** (R_6′_ = OH) exhibited 90.9% inhibition at 10 µM, being threefold more efficient than the corresponding methoxy analogue **25** (R_6′_ = OCH_3_; 33.3% inhibition). All these results showed the importance of the nature of the C-6′ substituent with the phenol function highly favourable for the best inhibitory potential. This parameter has to be combined with the substitution of the B ring by lipophilic atoms or groups. This B ring can be mono- or di-substituted without any impact on the inhibitory potential of the corresponding analogues. A similar trend was reported in a study comparing the inhibitory effect of 2′-hydroxy- and 2′-methoxychalcone on AR-dependent transcription in androgen-dependent LNCaP cells, with the phenolic derivatives being generally more active than or as active as their methoxylated counterparts [59].

Results from our preliminary screening at 10 µM allowed the selection of four active chalcones (**19**, **20**, **21**, and **23**) along with **18**, as the hit compound, and **25**, as a weak inhibitor. The 2,4-dichloro-2′,4′,6′-trihydroxychalcone **23** showed the most promising IC_50_ of 1.08 ± 0.27 µM (Table 1, Figure 2). Compounds **20** and **19** bearing a 3-methyl group and 4-methoxy group, respectively, were slightly less efficient but still with IC_50_ values in the low micromolar range (1.94 ± 0.32 µM and 2.36 ± 0.54 µM, respectively). Interestingly, shifting the methyl group from the C-4 position (**21**) to the C-3 position (**20**) led to an inhibitor almost threefold more potent, although both compounds displayed comparable inhibition in the previous screening at 10 µM (Table 1). Chalcone **25** exhibited a totally different profile from the other chalcones mentioned above. Interestingly, the inhibition went into saturation already at low compound concentrations without reaching 100% saturation. Therefore, determination of the IC_50_ value was not performed.

Docking simulations were then run, with all the chalcones listed in Table 1. Compounds which achieved a high score in the simulation and formed a hydrophobic contact with Tyr319 (compounds **19–23** and **26**) all showed a similar orientation within the binding pocket of the protein (Figure 6). The best inhibitor **23** additionally interacted with Tyr216 (Figure 6A). The C-4 methoxy group in compound **19** caused a shift of the molecule within the binding pocket moving ring B into subpocket SP3 where hydrogen bonds with Gln222 and NADP can be formed. This led to more potent inhibition of AKR1C3, compared with inhibition by **29** where only a C-4 hydroxy group is present. The C-6′ methoxy groups of **25** and **28** led to a positioning outside the binding pocket, explaining the low inhibitory activities of the compounds in enzymatic assays and the failure of **25** to reach 100% AKR1C3 inhibition in IC_50_ measurements. In contrast, **22** and **23** possessing a C-6′ hydroxy group were docked within the binding pocket and were potent inhibitors (Figure 6B). Hydrogen bonds with the catalytically important amino acids Tyr55 and His117, as well as the water molecules HOH 340, HOH 341, HOH 347, HOH 366, HOH 378, and/or HOH 468, were formed but could not be used to distinguish between active and inactive compounds.

While lipophilic substituents at the C-4 position enhanced AKR1C3 inhibition, the substitution with a methoxy group at position C-6′ on ring A strongly reduced, or even compromised, the inhibitory potency. As a methoxy residue is bulkier than a hydroxy group, this may lead to steric issues, but it can also lead to a strong intramolecular H bond between the C-2 phenol function and the carbonyl group of the chalcone core. As a consequence, the C-2 phenolic proton is less available for stabilising interaction with His117. The combination of these two parameters may explain the loss of affinity to the AKR1C3-binding site, as observed in the docking experiments and confirmed by enzymatic screening at 10 µM for inactive chalcones **24**, **25**, **27**, and **28**.

In the search for AKR1C3 inhibitors, many steroidal and non-steroidal inhibitors have been isolated or generated and analysed [16,29]. Among them, plant-derived chalcones, flavonoids, alkaloids, and/or their derivatives have also been investigated. One of these studies focused especially on the inhibitory influence of a series of naturally occurring chalcones on 17β-HSD and aromatase activities and was performed using human placental microsomes [47]. We included 2 of the 11 investigated chalcones—namely, isoliquiritigenin (**16**) and naringenin chalcone (**29**), in our study. Using placental microsomes and compound concentration of 10 µM, the authors reported 17β-HSD inhibition of 10% (**16**) and 2.4% (**29**), respectively. This is less than what was measured in our study, with **16** and **29** reaching 31.1 ± 3.2% and 40.6 ± 7.7% inhibition of AKR1C3, respectively. However, results cannot be compared directly because the assay setups differed, and unlike in our assay using bacterially overexpressed AKR1C3, an undefined mixture of 17β-HSDs was analysed with the placenta microsomes. In total, 10 17β-HSDs can be found in the human placenta [2], and microsome preparations may have depleted cytosolic AKR1C3, while primarily the membrane-bound 17β-HSDs [60] would have remained.

In addition to inhibitory potency, selectivity is an important feature in drug development. Selectivity analyses were thus performed by screening the most potent AKR1C3-inhibiting chalcones on putative off-targets, including the other enzymes of the AKR1C family and several 17β-HSDs. The activity of enzymes AKR1C1 and AKR1C4 was inhibited effectively by nearly all tested compounds at 10 µM. The inhibition seemed to be even stronger than that for AKR1C3, but this would have to be verified by IC_50_ analyses. AKR1C2 was inhibited as well by all compounds, albeit to a lesser extent. Only chalcone **23**, the best inhibitor of our chalcone series, strongly inhibited this enzyme. The low selectivity of the compounds is not very surprising, because it is very difficult to develop specific AKR1C isoform inhibitors due to the high homology (>84% of amino acids) between the human AKR1C enzymes, the very similar structures, and overlapping substrate preferences [6,34]. Indeed, other small molecules such as phytoestrogens of the flavonoid class or flufenamic acid can inhibit other AKR1C enzymes besides AKR1C3 and are thus not selective [30,45]. Chalcones are fairly small and might easily fit into the binding pockets of all AKR1C enzymes, although not all of these enzymes have cavities as large as that of AKR1C3 [30]. Bearing in mind the SAR results and the conclusions of the current work, more custom-fit chalcones could be designed in silico to better fill out the AKR1C3-binding cavity and its subpockets. The modification strategies applied to flufenamic acid [30] can be taken as a model.

It would be undesirable to inhibit the other isozymes of the AKR1C family when designing AKR1C3 inhibitors. AKR1C2 plays a key role in the inactivation of DHT to the non-potent AR ligand 3α,17β-androstanediol in the prostate [61]. Additionally, AKR1C1 can reduce DHT, in this case to 3β,17β-androstanediol, an ERβ ligand that acts as an antiproliferative and proapoptotic agent [62]. However, blocking DHT inactivation would mean enhancement of the AR pathway supporting cell proliferation. Additionally, by inhibiting the AKR1C1 enzyme, progesterone inactivation will be reduced, influencing the progesterone receptor pathway and steroid synthesis. Inhibition of the liver-specific AKR1C4 should also be avoided because this enzyme is also involved in the inactivation of DHT [6] and in the biosynthesis of bile acids [63,64].

Chalcones **18**–**21** and **23**, which efficiently targeted AKR1C3, also inhibited the steroid-metabolising 17β-HSDs of the short-chain dehydrogenase reductase family, although not as strongly as AKR1C enzymes. HSD17B1 converts estrone to oestradiol [65] and triggers the ER proliferative pathway [3]. Inhibition of this enzyme together with AKR1C3 would be beneficial for the treatment of diseases in which HSD17B1 is found to be upregulated [66]. HSD17B2 catalyses the oxidation of oestradiol to estrone and testosterone to ∆4-androstene-3,17-dione and has its role in eliminating active steroid hormones [2]. The enzyme counteracts the steroid-activating reactions of HSD17B1, HSD17B3, and AKR1C3, and inhibition of this enzyme would strongly affect steroid homeostasis. Inhibition of HSD17B3 would block testosterone synthesis in testis [2] which would be beneficial in case of desired testosterone deprivation, e.g., in prostate cancer treatment.

The six chalcones evaluated for selectivity were nearly ineffective on the SDR enzymes HSD17B4, HSD17B7, HSD17B10, and HSD17B14 (Figure 4C). Those enzymes can convert steroid hormones in vitro but are known (HSD17B4, HSD17B7, and HSD17B10) or postulated (HSD17B14) to be predominantly involved in other metabolic pathways in vivo [67,68,69,70]. For example, HSD17B7 is important in cholesterol synthesis, as it catalyses the conversion of zymosterone to zymosterol [68], and HSD17B4 plays its major role in the peroxisomal β-oxidation of VLCFA and branched-chain fatty acids [67]. The substrate-binding pockets of all these enzymes are very wide, in order to harbour the voluminous substrates [70], and chalcones are too small and might probably bind too loosely inside the pocket to be effective.

In the future, the panel of off-targets should be expanded. For example, Le Bail et al. showed that aromatase in human placental microsomes was inhibited with an IC_50_ of 2.6 µM by naringenin chalcone (**29**) [47]. Moreover, a clinical phase II trial for an endometriosis drug was stopped due to hepatotoxicity that was probably caused by inhibition of AKR1D1, important in bile acid metabolism [19]. Thus, both enzymes and several others [25] should be included in future in-depth selectivity assays.

In view of cancer treatment, cytotoxicity to target cells in addition to inhibition of AKR1C3 would be a valuable dual activity. However, normal cells should be as little affected as possible. The strong AKR1C3 inhibitors of our study were found to be cytotoxic only at high concentrations, if at all, to HEK293T cells which we took as a representative cell line for non-malignant cells. Interestingly, cell viability appeared to be higher than 100% at some concentrations when cells were treated with compounds **19**, **23**, or **25**. Whether this was due to the proliferative effects of the compounds on HEK293T cells or experimental variation is not clear. Surprisingly, chalcone **25**, a weak AKR1C3 inhibitor that could not be docked into the binding pocket of AKR1C3, had cytotoxic effects in almost the same concentration range as chemotherapeutic agents cisplatin and etoposide, conventionally used in cancer treatment. The underlying mechanism is unclear and would have to be analysed in future studies.

Although tested chalcones showed strong inhibition effects on the enzymes of the AKR1C family, the observed cytotoxicity of HEK293 cells cannot be attributed to this inhibition, because AKR1C enzymes are either absent or only negligibly expressed in HEK293 cells (UniProt database (https://www.uniprot.org, accessed on 5 January 2022)). Indeed, low endogenous expression was the reason to use this cell line for over-expression of AKR1C enzymes for inhibition screenings. Additionally, the HSD17B enzymes included in our screens were most probably not responsible for the cytotoxic effects. HSD17B2 and HSD17B3 are not present in HEK293 cells, and the weakly expressed HSD17B1 was not inhibited by **25**, the chalcone with the highest toxicity. Cytotoxicity of **25** can also not be explained by inhibition of HSD17B7, an enzyme expressed ubiquitously and needed for endogenous cholesterol synthesis, because impaired endogenous cholesterol supply should have been compensated by excess exogenous cholesterol in the culture medium. Thus, most probably, other targets in the cells are affected by the chalcones.

Additional studies are needed to clarify viability in disease cell models. As our study focused in the first place on optimisation of the AKR1C3 inhibitor scaffold, these analyses were not yet performed.

## 4. Materials and Methods

### 4.1. Synthesis of Chalcones

#### 4.1.1. General Procedures

All solvents were dried and distilled before use. Reactions were performed in an inert nitrogen atmosphere. Unless otherwise stated, materials purchased from commercial suppliers were used without further purification. ^1^H, ^13^C, and ^19^F-NMR spectra were recorded on a JEOL 400 MHz NMR spectrometer (JEOL USA Inc., Peabody, MA, USA) in deuterated solvents and calibrated using the residual undeuterated solvent resonance as an internal reference. Chemical shifts *δ* are given in ppm and coupling constants *J* in Hz. Mass spectrometry analyses were performed on a JMS-700 (JEOL USA Inc., Peabody, MA, USA) double-focusing mass spectrometer with reversed geometry, equipped with a pneumatically assisted ESI source. The reactions were monitored by analytical thin-layer chromatography using Polygram Sil G plate (Macherey-Nagel, Düren, Germany; silica gel 60 Å, 0.25 mm thick on an aluminium sheet). Column chromatography was performed by using silica gel 60 Å (particle size 40−63 µm) from Fisher Scientific. Flash chromatography purifications using prepacked columns (silica, 4 to 330 g) were carried out on a CombiFlash R_f_-200 apparatus equipped with a gradient pump, a column station with a DASi introduction system, a multiwavelength UV detector, a fraction collector, and appropriate software to control the device (Teledyne Isco, Lincoln, NE, USA). Isoliquiritigenin (**16**) and butein (**17**) were purchased from Aldrich (St. Louis, MO, USA).

##### General Procedure for Chalcone Formation

Method A: To a stirred solution of acetophenone **1** or **2** (1 mmol) in EtOH (3 mL) were added a selected benzaldehyde (1 mmol) and KOH (10 mmol). The reaction mixture was stirred at room temperature for 48 h, and then, ice-cold 10% aqueous HCl solution (20 mL) was added. Unless otherwise stated, the resulting mixture was extracted with EtOAc (3 × 10 mL), and combined organic layers were dried over anhydrous sodium sulphate, filtered, and concentrated under reduced pressure. The crude residue was purified by column chromatography on silica gel eluted with the appropriate mixture of solvents to afford the corresponding chalcone.

Method B: To a stirred solution of acetophenone **1** or **2** (1 mmol) in dioxane/water (1:1, 4 mL) were added a selected benzaldehyde (1 mmol) and NaOH (10 mmol). The reaction mixture was stirred at room temperature for 24 h, and then, ice-cold 10% aqueous HCl solution (20 mL) was added. The resulting mixture was extracted with EtOAc (3 × 10 mL), and combined organic layers were dried over anhydrous sodium sulphate, filtered, and concentrated under reduced pressure. The crude residue was purified by column chromatography on silica gel eluted with the appropriate mixture of solvents to afford the corresponding chalcone.

##### General Procedure for Phenol Deprotection

Method C: To a stirred solution of protected chalcone (1 mmol) in MeOH (30 mL) was added concentrated HCl (1.5 mL). The reaction mixture was stirred at room temperature for 40 h and then diluted with water (30 mL) and extracted with EtOAc (3 × 20 mL), and combined organic layers were dried over anhydrous sodium sulphate, filtered, and concentrated under reduced pressure. The residue was purified by column chromatography on silica gel eluted with the appropriate solvent to afford the desired products.

Method D: To a stirred solution of protected chalcone (1 mmol) in DCM/EtOH (1:7, 8 mL) at 0 °C was added *p*-toluenesulphonic acid monohydrate (10 mmol). The reaction mixture was stirred at 60 °C for 1 h and concentrated under reduced pressure. The mixture was then diluted with water (10 mL) and a saturated aqueous solution of NaHCO_3_ (10 mL) and extracted with EtOAc (3 × 20 mL). The combined organic layers were washed with brine (20 mL), dried over anhydrous sodium sulphate, filtered, and concentrated under reduced pressure. The residue was purified by column chromatography on silica gel eluted with the appropriate solvent to afford the desired products.

Further information on the synthesis of compounds of this study can be found in Supplementary Materials (Supplementary Chalcone Synthesis and Figures S2–S5).

### 4.2. Enzymatic Inhibition Assays

#### 4.2.1. Enzymes

For enzymatic assays and inhibitor testing, all enzymes were cloned into bacterial or mammalian expression vectors. AKR1C3 and the human hydroxysteroid dehydrogenases HSD17B1, HSD17B2, HSD17B4 (SDR domain), HSD17B7, and HSD17B10 were cloned into a pGEX vector as described in [71,72]. The coding sequences for AKR1C1, AKR1C2, and AKR1C4 were cloned by Gateway technology into the pcDNA3-N-MycDest vector according to the manufacturer’s instructions (Life Technologies, Carlsbad, CA, USA). The p11 plasmid coding for HSD17B14 (S205 variant) was a gift from Udo Opperman SGC Oxford, UK. For assays with HSD17B3, HEK293 cells stably expressing HSD17B3 were used [71]. Enzymes were expressed in *E. coli* BL21 DE3 or *E. coli* BL21 DE3 Codon Plus RP (Stratagene, San Diego, CA, USA) or in HEK293 cells as described in [71,72,73]. Bacterial and cell pellets were stored at −20 °C and −80 °C, respectively, until use.

#### 4.2.2. Inhibition Assays

Inhibition assays were carried out as described in [71,74]. Briefly, suspended bacteria, bacterial lysate, or suspended cells were incubated in an assay mixture containing sodium phosphate buffer, co-factor (600 µM NADPH or 750 µM NAD), 11.9 nM to 40 nM ^3^H-labelled steroid (PerkinElmer, Rodgau, Germany, or ARC, Saint Louis, MO, USA), and chalcone dissolved in DMSO (Merck Millipore, Darmstadt, Germany) in a final concentration of 10 µM for screenings or 0.1 µM to 50 µM (strong inhibitors) and 0.5 µM to 100 µM (weak inhibitors) for IC_50_ determination (1% DMSO final). For a detailed assay setup, see Supplementary Table S6. The incubation at 37 °C was stopped with 0.21 M ascorbic acid in methanol:acetic acid 99:1 (*v*:*v*) after the time needed to convert approximately 30% of the substrate in a control assay with 1% DMSO, without inhibitor candidate. Substrates and products were extracted from the reaction mixture by solid-phase extraction using C18 cartridges (Phenomenex, Aschaffenburg, Germany) and separated by RP–HPLC in a Beckman–Coulter HPLC Gold system, using the Luna 5 µm C18(2), 125 × 4.0 mm column (Phenomenex). The mobile phases used were H_2_O:ACN (57:43 (*v*:*v*); for assays of estrone (E1) to oestradiol (E2), E2 to E1, and ∆4-androstene-3,17-dione to testosterone conversion), H_2_O:ACN (49:51 (*v*:*v*); for assays of progesterone to 20α-hydroxyprogesterone conversion), and H_2_O:MeOH (35:65 (*v*:*v*) for assays of dihydrotestosterone (DHT) to 3α-androstanediol conversion) at a flow rate of 1 mL/min. Radioactivity was detected by online scintillation counting by using a radioactivity monitor (LB506D, Berthold Technologies, Bad Wildbad, Germany). Conversion of substrates to products was determined by integration of the peak area of the substrate and product peaks in percent. For calculation of inhibition, the conversion of control assay (assay without inhibitor) was set to 0% inhibition. All assays were run in triplicates, and mean values ± SD are reported.

For the determination of IC_50_ values, percent inhibition values were fitted using the Enzyme Kinetics module of SigmaPlot 13.0 and the one-site ligand-binding model according to the formula f = B_max_ * abs(x)/(K_d_ + abs(x)).

### 4.3. Cytotoxicity Assays

#### 4.3.1. Cell Culture

HEK293T cells were purchased from ATCC (CRL-3216). For the cultivation of HEK293T cells, Dulbecco’s modified Eagle medium (DMEM, Pan Biotech GmbH, Aidenbach, Germany) supplemented with 10% FCS (Pan Biotech GmbH), 100 U/mL penicillin, and 100 µg/mL streptomycin (Pan Biotech GmbH) was used. The cells were cultured at 37 °C and 5% CO_2_ in constant humidity. Before cell seeding, culture flasks and multiwell plates were coated with collagen G (0.001% in PBS, Merck, Darmstadt, Germany).

#### 4.3.2. Cytotoxicity Assays

For studying the cytotoxicity of the different chalcones and the positive control compounds cisplatin and etoposide on HEK293T cells, the Cell Titer^®^ Blue (CTB) Cell Viability Assay was used (Promega, Mannheim, Germany). A total of 10,000 cells in a 200 µL culture medium were seeded into each well of a 96-well plate. Then, 24 h after seeding, the cells were treated with 0, 1, 2.5, 5, 7.5, 10, 15, 25, 50, or 100 µM of the different compounds (3 wells per compound and concentration) and incubated for an additional 72 h. For measurement of cell viability, 20 µL of CTB reagent were added to each well, and after 5 h at 37 °C, the metabolic activity was quantified by recording the fluorescence signal (λ_Ex_ 560 nm, λ_Em_ 590 nm) on a Spark plate reader (Tecan Trading GmbH, Männedorf, Switzerland). Data are shown as mean values ± standard error of the mean (SEM). Statistical significances were calculated by one-way ANOVA (Dunnett’s multiple comparison test), * *p* < 0.05, ** *p* < 0.005, *** *p* < 0.0002, **** *p* < 0.0001.

### 4.4. Compound Docking and SAR Analysis

Molecular docking simulations were carried out on the crystal structure of the enzyme AKR1C3 co-crystallised with inhibitor 3-phenoxybenzoic acid (PDB Code: 3UWE, [75]) in GOLD version 5.2 and were analysed with LigandScout 4.4.5. (www.inteligand.com, accessed on 10 September 2021). All compounds were prepared for docking by energetically minimising them with omega 2.5.1.4. (OpenEye Scientific Software, Santa Fe, NM, USA) [76,77]. During protein preparation, hydrogens were added to the protein structure, and water molecule 341 was retained for docking. All remaining waters and the co-crystallised ligand were deleted. Atom CE1 of PHE306 was defined as the centre of the binding site, with a radius of 6 Å. GoldScore was used as a scoring function, and 10 docking poses were calculated for each ligand. For validation of the docking workflow, a re-docking was performed with an RMSD of 0.525 [78].

## 5. Conclusions

In the current study, structural optimisation of the hit backbone of natural chalcone **18** allowed the identification of various substituents that could be linked to a significantly improved inhibition of AKR1C3 activity. Among them, chalcones bearing a phloroglucinol ring showed better potency than their corresponding C-6′ methoxylated analogues. On ring B, lipophilic substituents were well tolerated, while the presence of a phenol function at the C-4 position systematically led to inactive derivatives. Substitution with OCH_3_, CH_3_, Cl, or F could be either in position C-4 or positions C-2 and C-4 of ring B. Combination of the optimal substitution parameters led to the identification of 2,4-dichloro-2′,4′,6′-trihydroxychalcone **23**, 10-fold more active than the natural chalcone **18**, the hit derivative from a previous study.

Docking poses of the most active chalcones confirm the importance of the C-6′phenolic function. These results bring new insights into the development of AKR1C3 inhibitors. Nevertheless, selectivity among the AKR1C family has not been successfully addressed. This is a major concern, and efforts are still required. Docking poses of **23** also showed that interaction with Tyr319 could be strengthened with bulkier substituents on ring B. Therefore, the current study paved the way to a new in silico design of chalcones, which could be led as a fragment growing approach starting from **23**, to further explore and develop AKR1C3 selective and efficient inhibitors.

## Figures and Tables

**Table 1:** Summary of chalcone backbone substitution pattern, inhibition of AKR1C3-catalysed ∆4-androstene-3,17-dione reduction, IC_50_ values for AKR1C3 inhibition, and results for compound docking into the AKR1C3-binding site.

	Substitution Pattern				AKR1C3 Inhibition		Docking and SAR Analyses	
Compound	R_6‘_	R_2_	R_3_	R_4_	Inhibition at 10 µM [%]	IC_50_ [µM]	Key Interactions	Docking Score
Isoliquiritigenin (**16**)	H	H	H	OH	31.1 ± 3.2	nd		65.81
Butein (**17**)	H	H	OH	OH	37.2 ± 1.4	nd		63.11
**18**	OCH_3_	H	H	OCH_3_	47.3 ± 6.4	11.91 ± 2.03		74.55
**19**	OH	H	H	OCH_3_	78.1 ± 2.7	2.36 ± 0.54	Tyr319	67.71
**20**	OH	H	CH_3_	H	89.2 ± 6.3	1.94 ± 0.32	Tyr319	69.80
**21**	OH	H	H	CH_3_	87.9 ± 4.9	5.18 ± 1.64	Tyr319	69.09
**22**	OH	H	H	Cl	70.8 ± 3.8	nd	Tyr319	66.90
**23**	OH	Cl	H	Cl	90.9 ± 4.6	1.08 ± 0.27	Tyr319, Tyr216	58.91
**24**	OCH_3_	H	H	OH	24.6 ± 4.8	nd	Tyr216	62.77
**25**	OCH_3_	Cl	H	Cl	33.3 ± 6.0	nd	out of binding pocket	48.26
**26**	OH	H	H	F	68.5 ± 6.4	nd	Tyr319	62.76
**27**	OCH_3_	H	H	F	20.3 ± 8.4	nd	out of binding pocket	45.19
**28**	OCH_3_	H	H	Cl	26.1 ± 2.0	nd	out of binding pocket	48.32
**29**	OH	H	H	OH	40.6 ± 7.7	nd		64.83

SAR—structure-activity relationship; nd—not determined.

## Data Availability

The data presented in this study can be found in this article and the supplement. Further information and requests for resources should be directed to and will be fulfilled by the corresponding author, Gabriele Möller (gabriele.moeller@helmholtz-muenchen.de).

## Supplementary Materials

The following supporting information can be downloaded at: https://www.mdpi.com/article/10.3390/metabo12020099/s1, Figure S1: Procedure for chalcone synthesis, Table S1: Chalcone substitution patterns, chalcone synthesis**,** Figure S2: ^1^H and ^13^C NMR spectra of **6** in CDCl_3_, Figure S3: ^1^H and ^13^C NMR spectra of **11** in acetone-*d*_6_, Figure S4: ^1^H and ^13^C NMR spectra of **20** in acetone-*d*_6_, Figure S5: ^1^H and ^13^C NMR spectra of **25** in acetone-*d*_6_, Table S2: Chalcone structures and inhibition data, Table S3: Compound selectivity—inhibition of human AKR1C enzymes by chalcones at 10 µM concentration in %, Table S4: Compound selectivity—inhibition of human SDR enzymes primarily involved in steroid metabolism by chalcones at 10 µM concentration in %, Table S5: Compound selectivity—inhibition of human SDR enzymes primarily involved in other pathways than steroid metabolism by chalcones at 10 µM concentration in %, Table S6: Activity assay setup.
