# Supplementary material for: Analogues of Natural Chalcones as Efficient Inhibitors of AKR1C3
Source: Metabolites. 2022 Jan 21;12(2):99. doi: 10.3390/metabo12020099 (PMC8876231; doi:10.3390/metabo12020099)
Supplement: Supplementary file 1 [file metabolites-12-00099-s001.zip › metabolites-1545010-supplementary.pdf]

Supplementary Information

Analogues of natural chalcones as efficient inhibitors of AKR1C3

Gabriele Möller[1], Veronika Temml[2], Antonio C. Peralta[3], Océane Gruet[3], Pascal Richomme[3], Denis Séraphin[3], Guillaume Viault[3], Luisa Kraus[4], Petra Huber-Cantonati[4], Elisabeth Schopfhauser[2], Johanna Pachmayr[4], Janina Tokarz[1], Daniela Schuster[2], Jean-Jacques Helesbeux[3] and Kenneth A. Dyar[1]

[1] Metabolic Physiology, Institute for Diabetes and Cancer, Helmholtz Center Munich, German Research Center for Environmental Health, Neuherberg, Germany
[2] Institute of Pharmacy, Department of Pharmaceutical and Medicinal Chemistry, Paracelsus Medical University Salzburg, Salzburg, Austria
[3] Univ Angers, SONAS, SFR QUASAV, F-49000, Angers, France
[4] Institute of Pharmacy, Pharmaceutical Biology and Clinical Pharmacy, Paracelsus Medical University Salzburg, Salzburg, Austria

Content

Figure S1: Procedure for chalcone synthesis — Page 2

Table S1: Chalcone substitution patterns — Page 2

Chalcone Synthesis — Page 3 – 11

Figure S2: ^{1}H and ^{13}C NMR spectra of **6** in $CDCl_3$ — Page 12

Figure S3: ^{1}H and ^{13}C NMR spectra of **11** in acetone-d_6 — Page 13

Figure S4: ^{1}H and ^{13}C NMR spectra of **20** in acetone-d_6 — Page 14

Figure S5: ^{1}H and ^{13}C NMR spectra of **25** in acetone-d_6 — Page 15

Table S2: Chalcone structures and inhibition data. — Page 16

Table S3: Compound selectivity - Inhibition of human AKR1C enzymes by chalcones at 10 µM concentration in % — Page 17

Table S4: Compound selectivity - Inhibition of human SDR enzymes primarily involved in steroid metabolism by chalcones at 10 µM concentration in % — Page 17

Table S5: Compound selectivity - Inhibition of human SDR enzymes primarily involved in other pathways than steroid metabolism by chalcones at 10 µM concentration in % — Page 17

Table S6: Activity assay setup — Page 18

References — Page 18 – 19

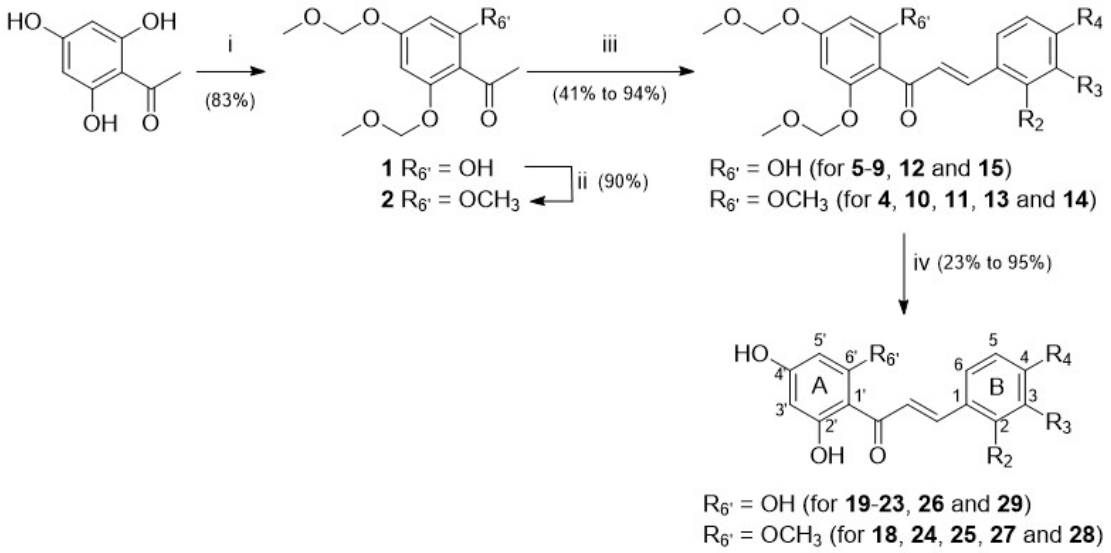

Figure S1: Procedure for chalcone synthesis

Table S1: Chalcone substitution patterns

**Chalcone**	**$R_{6'}$**	**R_2**	**R_3**	**R_4**
Isoliquiritigenin (**16**)	H	H	H	OH
Butein (**17**)	H	H	OH	OH
**18**	OCH_3	H	H	OCH_3
**19**	OH	H	H	OCH_3
**20**	OH	H	CH_3	H
**21**	OH	H	H	CH_3
**22**	OH	H	H	Cl
**23**	OH	Cl	H	Cl
**24**	OCH_3	H	H	OH
**25**	OCH_3	Cl	H	Cl
**26**	OH	H	H	F
**27**	OCH_3	H	H	F
**28**	OCH_3	H	H	Cl
**29**	OH	H	H	OH

Chalcone Synthesis

General information

All solvents were dried and distilled before use. Reactions were performed in an inert nitrogen atmosphere. Unless otherwise stated, materials purchased from commercial suppliers were used without further purification. ^{1}H, ^{13}C and ^{19}F-NMR spectra were recorded on a JEOL 400 MHz NMR spectrometer (JEOL USA Inc, USA) in deuterated solvents and calibrated using the residual undeuterated solvent resonance as internal reference. Chemical shifts δ are given in ppm and coupling constants J in Hz. Mass spectrometry analyses were performed on a JMS-700 (JEOL USA Inc, USA) double-focusing mass spectrometer with reversed geometry, equipped with a pneumatically assisted ESI source. The reactions were monitored by analytical thin layer chromatography using Polygram Sil G plate (Macherey-Nagel; silica gel 60 Å, 0.25 mm thick on aluminium sheet). Column chromatography was performed by using silica gel 60 Å (particle size 40−63 µm) from Fisher Scientific. Flash chromatography purifications using prepacked columns (silica, 4 to 330 g) were carried out on a CombiFlash R_f-200 apparatus equipped with a gradient pump, a column station with a DASi introduction system, a multiwavelength UV detector, a fraction collector, and appropriate software to control the device (Teledyne Isco, USA). Isoliquiritigenin (**16**) and butein (**17**) were purchased from Aldrich.

General synthetic procedure for chalcone formation

Method A: To a stirred solution of acetophenone **1** or **2** (1 mmol) in EtOH (3 mL) were added a selected benzaldehyde (1 mmol) and KOH (10 mmol). The reaction mixture was stirred at room temperature for 48 h and then ice-cold 10 % aqueous HCl solution (20 mL) was added. Unless otherwise stated, the resulting mixture was extracted with EtOAc (3 x 10 mL) and combined organic layers were dried over anhydrous sodium sulfate, filtered and concentrated under reduced pressure. The crude residue was purified by column chromatography on silica gel eluted with the appropriate mixture of solvents to afford the corresponding chalcone.

Method B: To a stirred solution of acetophenone **1** or **2** (1 mmol) in dioxane/water (1:1, 4 mL) were added a selected benzaldehyde (1 mmol) and NaOH (10 mmol). The reaction mixture was stirred at room temperature for 24 h and then ice-cold HCl 10 % aqueous HCl solution (20 mL) was added. The resulting mixture was extracted with EtOAc (3 x 10 mL) and combined organic layers were dried over anhydrous sodium sulfate, filtered and concentrated under reduced pressure. The crude residue was purified by column chromatography on silica gel eluted with the appropriate mixture of solvents to afford the corresponding chalcone.

General synthetic procedure for phenol deprotection

Method C: To a stirred solution of protected chalcone (1 mmol) in MeOH (30 mL) was added concentrated HCl (1.5 mL). The reaction mixture was stirred at room

temperature for 40 h and then diluted with water (30 mL) and extracted with EtOAc (3 x 20 mL) and combined organic layers were dried over anhydrous sodium sulfate, filtered and concentrated under reduced pressure. The crude residue was purified by column chromatography or preparative TLC on silica gel eluted with the appropriate mixture of solvents to afford the expected chalcone.

Method D: To a stirred solution of protected chalcone (1 mmol) in DCM/EtOH (1:7, 8 mL) at 0 °C was added *p*-toluenesulfonic acid monohydrate (10 mmol). The reaction mixture was stirred at 60 °C for 1 h and concentrated under reduced pressure. The mixture was then diluted with 20 mL of half-saturated aqueous solution of $NaHCO_3$ (10 mL) and extracted with AcOEt (3 x 20 mL). The combined organic layers were washed with brine (20 mL), dried over anhydrous sodium sulfate, filtered and concentrated under reduced pressure. The crude residue was purified by column chromatography or preparative TLC on silica gel eluted with the appropriate mixture of solvents to afford the expected chalcone.

1-(2-hydroxy-4,6-bis(methoxymethoxy)phenyl)ethanone **1** [1].

To a solution of 2′,4′,6′-trihydroxyacetophenone (98%, 3 g, 17.85 mmol) in dry THF (100 mL), *N*,*N*-diisopropylethylamine (9.5 mL, 3 eq.) and bromomethyl methyl ether (4.4 mL, 3 eq.) were added at 0 °C under N_2 atmosphere. The reaction mixture was stirred at room temperature for 2 h then quenched by adding 80 mL of ice-cold water and pH was lowered to 4 with 10 % aqueous HCl solution. The mixture was extracted with EtOAc (3 x 100 mL) and combined organic layers were dried over anhydrous sodium sulfate, filtered and concentrated under reduced pressure. The residue was purified by column chromatography on silica gel eluted with petroleum ether:acetone mixture (9:1), to afford compound **1** (3.8 g, 83% yield,) as a colorless solid. R_f = 0.4 (PE/EtOAc, 4:1); ^{1}H NMR (400 MHz, $CDCl_3$) δ 13.72 (s, 1H), 6.26 (d, *J* = 2.1 Hz, 1H), 6.24 (d, *J* = 2.1 Hz, 1H), 5.25 (s, 2H), 5.17 (s, 2H), 3.51 (s, 3H), 3.47 (s, 3H), 2.65 (s, 3H); ^{13}C NMR (100 MHz, $CDCl_3$) δ 203.4, 166.9, 163.6, 160.5, 107.0, 97.2, 94.6, 94.1 (2C), 56.8, 56.6, 33.2.

1-(2-methoxy-4,6-bis(methoxymethoxy)phenyl)ethanone **2** [2].

To a solution of 1-(2-hydroxy-4,6-bis(methoxymethoxy)phenyl)ethanone **1** (2 g, 7.81 mmol) in dry DMF (25 mL), sodium hydride 60% in mineral oil (406 mg, 10.15 mmol) and iodomethane (834 µL, 13.27 mmol) were added at 0 °C under N_2 atmosphere. The reaction mixture was stirred at room temperature for 1 h then quenched by ice cold H_2O (50 mL) and pH was lowered to 7 with 10 % HCl. The mixture was extracted with Et_2O (3 x 50 mL) and combined organic layers were dried over anhydrous sodium sulfate, filtered and concentrated under reduced pressure to afford acetophenone **2** (1.89 g, 90% yield,) as a colorless solid. The crude product was used in the next step without further purification. R_f = 0.5 (PE/EtOAc, 4:1); ^{1}H NMR (400 MHz, acetone-d_6) δ 6.48 (d, *J* = 1.5 Hz, 1H), 6.41 (d, *J* = 1.5 Hz, 1H), 5.21 (s, 2H), 5.16

(s, 2H), 3.79 (s, 3H), 3.44 (s, 3H), 3.41 (s, 3H), 2.36 (s, 3H); ^{13}C NMR (100 MHz, acetone-d_6) δ 200.3, 160.4, 158.5, 155.8, 116.9, 96.8, 95.5, 95.1, 94.7, 56.4, 56.3, 56.2, 32.6.

4-(methoxymethoxy)benzaldehyde **3** [3].
To a solution of 4-hydroxybenzaldehyde (1 g, 8.19 mmol) in dry THF (20 mL), *N*,*N*-diisopropylethylamine (4.3 mL, 24.5 mmol) and bromomethyl methyl ether (1.1 mL, 12.1 mmol) were added at 0 °C under N_2 atmosphere. The reaction mixture was stirred at room temperature for 21 h then quenched by adding 20 mL of ice-cold water and pH was lowered to 4 with 10 % aqueous HCl. The resulting mixture was extracted with EtOAc (3 x 25 mL). The combined organic layers were dried over anhydrous sodium sulfate, filtered and concentrated under reduced pressure to afford compound **3** (1.35 g, 99% yield,) as a brown oil. R_f = 0.4 (PE/EtOAc, 4:1); ^{1}H NMR (400 MHz, $CDCl_3$) δ 9.87 (s, 1H), 7.81 (d, *J* = 8.6 Hz, 2H), 7.12 (d, *J* = 8.6 Hz, 2H), 5.23 (s, 2H), 3.46 (s, 3H); ^{13}C NMR (100 MHz, $CDCl_3$) δ 191.0, 162.3, 132.0 (2C), 130.7, 116.3 (2C), 94.1, 56.4.

(*E*)-1-(2-methoxy-4,6-bis(methoxymethoxy)phenyl)-3-(4-methoxyphenyl)prop-2-en-1-one **4** [4].
This chalcone was synthesized following the general method A for chalcone formation, starting from acetophenone **2** and 4-methoxybenzaldehyde. Chalcone **4** was obtained as a yellow oil, with 80% yield. R_f = 0.3 (PE/Acetone, 7:3); ^{1}H NMR (400 MHz, acetone-d_6) δ 7.60 (d, *J* = 8.8 Hz, 2H), 7.27 (d, *J* = 16.1 Hz, 1H), 6.97 (d, *J* = 8.8 Hz, 2H), 6.83 (d, *J* = 16.1), 6.53 (d, *J* = 2.0 Hz, 1H), 6.46 (d, *J* = 2.0, 1H), 5.24 (s, 2H), 5.12 (s, 2H), 3.84 (s, 3H), 3.74 (s, 3H), 3.47 (s, 3H), 3.34 (s, 3H); ^{13}C NMR (100 MHz, acetone-d_6) δ 193.6, 162.5, 160.5, 159.2, 156.5, 144.7, 130.9 (2C), 128.4, 127.9, 115.3 (2C), 115.0, 96.7, 95.3, 95.2, 94.8, 56.34, 56.29, 56.2, 55.8.

(*E*)-1-(2-hydroxy-4,6-bis(methoxymethoxy)phenyl)-3-(4-methoxyphenyl)prop-2-en-1-one **5** [5].
This chalcone was synthesized following the method A for chalcone formation, starting from acetophenone **1** and 4-methoxybenzaldehyde. Chalcone **5** was obtained as a yellow oil, with 94% yield. R_f = 0.3 (PE/Acetone, 8:2); ^{1}H NMR (400 MHz, $CDCl_3$) δ 13.95 (s, 1H, OH), 7.96 (d, *J* = 15.6 Hz, 1H), 7.79 (d, *J* = 15.6 Hz, 1H), 7.71 (d, *J* = 8.7 Hz, 2H), 7.02 (d, *J* = 8.7 Hz), 6.32 (d, *J* = 2.3 Hz, 1H), 6.25 (d, *J* = 2.3 Hz, 1H), 5.43 (s, 2H), 5.26 (s, 2H) , 3.87 (s, 3H), 3.55 (s, 3H), 3.46 (s, 3H); ^{13}C NMR (100 MHz, $CDCl_3$) δ 193.6, 168.1, 164.5, 162.7, 161.0, 143.6, 131.2 (2C), 128.9, 125.6, 115.4 (2C), 108.0, 97.8, 96.1, 95.5, 94.9, 57.1, 56.5, 55.8.

(*E*)-1-(2-hydroxy-4,6-bis(methoxymethoxy)phenyl)-3-(*m*-tolyl)prop-2-en-1-one **6**.

This chalcone was synthesized following the method A for chalcone formation, starting from acetophenone **1** and 3-methylbenzaldehyde. Chalcone **6** was obtained as a yellow oil, with 89% yield. R_f = 0.2 (PE/Acetone, 9:1); IR (ν cm^{-1}): 1624, 1585, 1240, 1150, 1055, 1022; ^{1}H NMR (400 MHz, $CDCl_3$) δ 13.88 (s, 1H, OH), 7.92 (d, *J* = 15.6 Hz, 1H), 7.75 (d, *J* = 15.6 Hz, 1H), 7.41 (d, *J* = 7.5 Hz, 1H), 7.40 (bs, 1H), 7.29 (t, *J* = 7.5 Hz, 1H), 7.20 (d, *J* = 7.5 Hz, 1H), 6.31 (d, *J* = 2.4 Hz, 1H), 6.24 (d, *J* = 2.4 Hz, 1H), 5.29 (s, 2H), 5.18 (s, 2H), 3.54 (s, 3H), 3.48 (s, 3H), 2.38 (s, 3H); ^{13}C NMR (100 MHz, $CDCl_3$) δ 193.0, 167.4, 163.5, 159.9, 142.8, 138.6, 135.4, 131.1, 129.2, 128.9, 127.2, 125.4, 107.5, 97.5, 95.2, 94.7, 94.1, 57.0, 56.5, 21.4. HRMS (ESI) m/z calcd for $C_{20}H_{21}O_6$ $[M-H]^-$ 357.1338, found 357.1342.

(*E*)-1-(2-hydroxy-4,6-bis(methoxymethoxy)phenyl)-3-(*p*-tolyl)prop-2-en-1-one **7** [5].
This product was synthesized following the method A for chalcone formation, starting from acetophenone **1** and 4-methylbenzaldehyde. Chalcone **7** was obtained through filtration after water washes as an orange solid, with 84% yield. R_f = 0.2 (PE/Acetone, 9:1); ^{1}H NMR (400 MHz, acetone-d_6) δ 13.88 (s, 1H), 8.02 (d, *J* = 15.6 Hz, 1H), 7.77 (d, *J* = 15.6 Hz, 1H), 7.63 (d, *J* = 8.0 Hz, 2H), 7.27 (d, *J* = 8.0 Hz, 2H), 6.32 (d, *J* = 2.4 Hz, 1H), 6.26 (d, *J* = 2.4 Hz, 1H), 5.42 (s, 2H), 5.26 (s, 2H), 3.55 (s, 3H), 3.46 (s, 3H), 2.37 (s, 3H); ^{13}C NMR (100 MHz, acetone-d_6) δ 193.7, 168.1, 164.6, 161.0, 143.5, 141.6, 133.6, 130.6 (2C), 129.4 (2C), 127.2, 108.0, 97.7, 96.1, 95.6, 94.9, 57.1, 56.6, 21.5.

(*E*)-3-(4-chlorophenyl)-1-(2-hydroxy-4,6-bis(methoxymethoxy)phenyl)prop-2-en-1-one **8** [5].
This product was synthesized following the method A for chalcone formation, starting from acetophenone **1** and 4-chlorobenzaldehyde. Chalcone **8** was obtained through filtration after water washes as an orange solid, with 78% yield. R_f = 0.2 (PE/Acetone, 9:1); ^{1}H NMR (400 MHz, acetone-d_6) δ 13.76 (s, 1H), 8.04 (d, *J* = 15.7 Hz, 1H), 7.75 (d, *J* = 8.3 Hz, 2H), 7.74 (d, *J* = 15.7 Hz, 1H), 7.27 (d, *J* = 8.3 Hz, 2H), 6.32 (d, *J* = 2.3 Hz, 1H), 6.26 (d, *J* = 2.3 Hz, 1H), 5.42 (s, 2H), 5.27 (s, 2H), 3.54 (s, 3H), 3.46 (s, 3H); ^{13}C NMR (100 MHz, acetone-d_6) δ 193.5, 168.1, 164.8, 161.1, 141.5, 136.3, 135.2, 130.8 (2C), 130.0 (2C), 129.0, 107.9, 97.7, 96.1, 95.6, 94.9, 57.1, 56.6.

(*E*)-3-(2,4-dichlorophenyl)-1-(2-hydroxy-4,6-bis(methoxymethoxy)phenyl)prop-2-en-1-one **9** [5].
This chalcone was synthesized following the method A for chalcone formation, starting from acetophenone **1** and 2,4-dichlorobenzaldehyde. Chalcone **9** was obtained through filtration after water washes as an orange solid, with 71% yield. R_f = 0.2 (PE/Acetone, 9:1); ^{1}H NMR (400 MHz, $CDCl_3$) δ 13.72 (s, 1H), 8.04 (d, *J* = 15.6 Hz, 1H), 7.87 (d, *J* = 15.6 Hz, 1H), 7.60 (d, *J* = 8.5 Hz, 1H), 7.44 (d, *J* = 2.1 Hz, 1H), 7.27 (dd, *J* = 2.1, 8.5 Hz, 1H), 6.30 (d, *J* = 2.3 Hz, 1H), 6.22 (d, *J* = 2.3 Hz, 1H), 5.27 (s, 2H),

5.18 (s, 2H), 3.50 (s, 3H), 3.48 (s, 3H); ^{13}C NMR (100 MHz, $CDCl_3$) δ 192.4, 167.6, 163.9, 159.9, 136.7, 136.1, 136.0, 132.4, 130.3, 130.2, 128.5, 127.6, 107.4, 97.6, 95.3, 94.8, 94.1, 57.0, 56.6.

(*E*)-1-(2-methoxy-4,6-bis(methoxymethoxy)phenyl)-3-(4-(methoxymethoxy)phenyl)prop-2-en-1-one **10** [6].

This chalcone was synthesized following the method B for chalcone formation, starting from acetophenone **2** and benzaldehyde **3**. Chalcone **10** was obtained as a yellow oil, with 46% yield. R_f = 0.2 (PE/Acetone, 9:1); ^{1}H NMR (400 MHz, $CDCl_3$) δ 7.44 (d, *J* = 8.6 Hz, 2H), 7.29 (d, *J* = 16.1 Hz, 1H), 6.99 (d, *J* = 8.8 Hz, 2H), 6.83 (d, *J* = 16.1 Hz, 1H), 6.48 (d, *J* = 2.0 Hz, 1H), 6.34 (d, *J* = 2.0 Hz, 1H), 5.17 (s, 2H), 5.16 (s, 2H), 5.08 (s, 2H), 3.72 (s, 3H), 3.48 (s, 3H), 3.43 (s, 3H), 3.35 (s, 3H); ^{13}C NMR (100 MHz, $CDCl_3$) δ 194.6, 159.8, 159.1, 158.5, 155.9, 144.8, 130.1 (2C), 128.6, 127.4, 116.5 (2C), 113.9, 95.9, 94.6, 94.2, 94.0, 56.4, 56.3, 56.2, 56.0, 31.0.

(*E*)-3-(2,4-dichlorophenyl)-1-(2-methoxy-4,6-bis(methoxymethoxy)phenyl)prop-2-en-1-one **11**.

This chalcone was synthesized following the method B for chalcone formation, starting from acetophenone **2** and 2,4-dichlorobenzaldehyde. Chalcone **11** was obtained as a yellow oil, with 92% yield. R_f = 0.2 (PE/Acetone, 9:1); IR (ν cm^{-1}): 2950, 2829, 1654, 1601, 1463, 1397, 1224, 1148, 1116, 1076, 1017, 920, 822; ^{1}H NMR (400 MHz, acetone-d_6) δ 7.95 (d, *J* = 8.4 Hz, 1H), 7.66 (d, *J* = 16.1 Hz , 1H), 7.58 (s, 1H), 7.45 (d, *J* = 8.5 Hz, 1H), 7.02 (d, *J* = 16.1 Hz, 1H), 6.55 (s, 1H), 6.48 (s, 1H), 5.25 (s, 2H), 5.15 (s, 2H), 3.78 (s, 3H), 3.47 (s, 3H), 3.37 (s, 3H); ^{13}C NMR (100 MHz, acetone-d_6) δ 193.0, 161.0, 159.5, 156.9, 138.0, 136.7, 135.9, 132.8, 130.5, 130.1, 128.8, 127.1, 114.4, 96.8, 95.5, 95.2, 94.7, 56.4, 56.4, 56.3; HRMS (ES) m/z calcd for $C_{20}H_{20}O_6Na$ $[M+Na]^+$ 449.0535 found 449.0533.

(*E*)-3-(4-fluorophenyl)-1-(2-hydroxy-4,6-bis(methoxymethoxy)phenyl)prop-2-en-1-one **12** [5].

This chalcone was synthesized following the method B for chalcone formation, starting from acetophenone **1** and 4-fluorobenzaledhyde. Chalcone **12** was obtained as a yellow oil, with 80% yield. R_f = 0.2 (PE/Acetone, 9:1); ^{1}H NMR (400 MHz, $CDCl_3$) δ 13.84 (s, 1H), 7.82 (d, *J* = 15.6 Hz, 1H), 7.68 (d, *J* = 15.6 Hz, 1H), 7.54 (dd, *J* = 8.6, 5.4 Hz, 2H), 7.05 (t, *J* = 8.6 Hz, 2H), 6.27 (d, *J* = 2.3 Hz, 1H), 6.20 (d, *J* = 2.3 Hz, 1H), 5.26 (s, 2H), 5.15 (s, 2H), 3.50 (s, 3H), 3.45 (s, 3H); ^{13}C NMR (100 MHz, $CDCl_3$) δ 192.7, 167.5, 163.9 (d, $^1J_{C-F}$ = 251 Hz), 163.6, 159.9, 141.2, 131.8 (d, $^4J_{C-F}$ = 3,3 Hz), 130.2 (d, $^3J_{C-F}$ = 8,4 Hz, 2C), 127.1 (d, $^5J_{C-F}$ = 2.2 Hz), 116.1 (d, $^2J_{C-F}$ = 21.9 Hz, 2C), 107.4, 97.6, 95.2, 94.8, 94.1, 57.0, 56.5; ^{19}F NMR (376 MHz, $CDCl_3$) δ -108.5.

(E)-3-(4-fluorophenyl)-1-(2-methoxy-4,6-bis(methoxymethoxy)phenyl)prop-2-en-1-one **13** [7].
This chalcone was synthesized following the method B for chalcone formation, starting from acetophenone **2** and 4-fluorobenzaldehyde. Chalcone **13** was obtained as an orange oil, with 46% yield. R_f = 0.2 (PE/Acetone, 9:1); ^{1}H NMR (400 MHz, $CDCl_3$) δ 7.49 (dd, *J* = 8.6, 5.4 Hz, 2H), 7.31 (d, *J* = 16.1 Hz, 1H), 7.03 (t, *J* = 8.6 Hz, 2H), 6.87 (d, *J* = 16.1 Hz, 1H), 6.49 (d, *J* = 2.0 Hz, 1H), 6.35 (d, *J* = 2.0 Hz, 1H), 5.17 (s, 2H), 5.09 (s, 2H), 3.74 (s, 3H), 3.48 (s, 3H), 3.36 (s, 3H); ^{13}C NMR (100 MHz, $CDCl_3$) δ 194.1, 163.9 (d, $^1J_{C-F}$ = 251.5 Hz), 160.0, 158.6, 155.9, 143.4, 131.1 (d, $^4J_{C-F}$ = 3.3 Hz), 130.3 (d, $^3J_{C-F}$ = 8.5 Hz, 2C), 128.7 (d, $^5J_{C-F}$ = 2.2 Hz), 116.0 (d, $^2J_{C-F}$ = 21.9 Hz, 2C), 113.6, 95.9, 94.6, 94.5, 94.0, 56.3, 56.3, 56.0; ^{19}F NMR (376 MHz, $CDCl_3$) δ -111.7.

(*E*)-3-(4-chlorophenyl)-1-(2-methoxy-4,6-bis(methoxymethoxy)phenyl)prop-2-en-1-one **14** [4].
This chalcone was synthesized following the method B for chalcone formation, starting from acetophenone **2** and 4-chlorobenzaldehyde. Chalcone **14** was obtained as a yellow oil, with 47% yield. R_f = 0.2 (PE/Acetone, 85:15); ^{1}H NMR (400 MHz, $CDCl_3$) δ 7.41 (d, *J* = 8.4 Hz, 2H), 7.33 – 7.25 (m, 3H), 6.89 (d, *J* = 16.1 Hz, 1H), 6.47 (d, *J* = 2.0 Hz, 1H), 6.33 (d, *J* = 2.0 Hz, 1H), 5.15 (s, 2H), 5.07 (s, 2H), 3.72 (s, 3H), 3.46 (s, 3H), 3.34 (s, 3H); ^{13}C NMR (100 MHz, $CDCl_3$) δ 194.1, 160.1, 158.6, 156.1, 143.1, 136.2, 133.5, 129.6 (2C), 129.4, 129.2 (2C), 113.6, 95.9, 94.7, 94.6, 94.0, 56.4, 56.4, 56.0.

(*E*)-1-(2-hydroxy-4,6-bis(methoxymethoxy)phenyl)-3-(4-(methoxymethoxy)phenyl)prop-2-en-1-one **15** [8].
This chalcone was synthesized following the method B for chalcone formation, starting from acetophenone **1** and benzaldehyde **3**. Chalcone **15** was obtained as a yellow oil, with 41% yield. R_f = 0.2 (PE/Acetone, 9:1); ^{1}H NMR (400 MHz, acetone-d_6) δ 13.91 (s, 1H), 7.82 (d, *J* = 15.6 Hz, 1H), 7.75 (d, *J* = 15.6 Hz, 1H), 7.53 (d, *J* = 8.6 Hz, 2H), 7.04 (d, *J* = 8.8 Hz, 2H), 6.29 (d, *J* = 2.4 Hz, 1H), 6.22 (d, *J* = 2.4 Hz, 1H), 5.27 (s, 2H), 5.19 (s, 2H), 5.16 (s, 2H), 3.52 (s, 3H), 3.47 (s, 3H), 3.46 (s, 3H); ^{13}C NMR (100 MHz, acetone-d_6) δ 192.9, 167.4, 163.4, 159.9, 159.1, 142.5, 130.1 (2C), 129.3, 125.5, 116.6 (2C), 107.6, 97.5, 95.2, 94.8, 94.3, 94.1, 57.0, 56.5, 56.3.

(*E*)-1-(2,4-dihydroxy-6-methoxyphenyl)-3-(4-methoxyphenyl)prop-2-en-1-one **18** [4].
This chalcone was synthesized following the method D for phenol deprotection starting from **4** and was obtained with 78% yield, as a yellow oil. R_f = 0.2 (PE/Acetone, 8:2); ^{1}H NMR (400 MHz, acetone-d_6) δ 14.30 (bs, 1H, OH), 9.44 (bs, 1H, OH), 7.91 (d, *J* = 15.6 Hz, 1H), 7.75 (d, *J* = 15.6 Hz, 1H), 7.69 (d, *J* = 8.8 Hz, 2H), 7.01 (d, *J* = 8.8 Hz, 2H), 6.07 (d, *J* = 2.3 Hz, 1H), 5.99 (d, *J* = 2.3 Hz, 1H), 3.98 (s, 3H), 3.86 (s,

3H); ^{13}C NMR (100 MHz, acetone-d_6) δ 193.0, 169.0, 165.7, 164.2, 162.5, 142.9, 131.0 (2C), 129.0, 125.9, 115.2 (2C), 106.3, 96.9, 92.1, 56.3, 55.7.

(*E*)-3-(4-methoxyphenyl)-1-(2,4,6-trihydroxyphenyl)prop-2-en-1-one **19** [5].
This chalcone was synthesized following the method C for phenol deprotection starting from **5** and was obtained with 38% yield, as a yellow oil. R_f = 0.2 (DCM/MeOH, 95:5); ^{1}H NMR (400 MHz, acetone-d_6) δ 8.19 (d, *J* = 15.6 Hz, 1H), 7.76 (d, *J* = 15.6 Hz, 1H), 7.64 (d, *J* = 8.8 Hz, 2H), 6.99 (d, *J* = 8.8 Hz, 2H), 5.97 (s, 2H), 3.85 (s, 3H); ^{13}C NMR (100 MHz, acetone-d_6) δ 193.1, 165.9 (2C), 165.8, 162.4, 142.6, 130.9 (2C), 129.1, 126.1, 115.3 (2C), 105.7, 96.0 (2C), 55.7.

(*E*)-3-(*m*-tolyl)-1-(2,4,6-trihydroxyphenyl)prop-2-en-1-one **20**.
This chalcone was synthesized following the method C for phenol deprotection starting from **6** and was obtained with 78% yield, as an orange solid. R_f = 0.4 (PE/Acetone, 7:3); IR (ν cm^{-1}): 3257, 2923, 2853, 1625, 1510, 1457, 1338, 1241, 1165, 1079, 1027, 826; ^{1}H NMR (400 MHz, acetone-d_6) δ 12.00 (bs, 2H), 9.36 (bs, 1H), 8.24 (d, *J* = 15.6 Hz, 1H), 7.75 (d, *J* = 15.6 Hz, 1H), 7.50 (bs, 1H), 7.48 (d, *J* = 7.6 Hz, 1H), 7.32 (t, *J* = 7.6 Hz, 1H), 7.24 (d, *J* = 7.6 Hz, H1), 5.98 (s, 2H), 2.36 (s, 3H); ^{13}C NMR (100 MHz, acetone-d_6) δ 193.2, 165.7 (3C), 142.8, 139.4, 136.5, 131.7, 129.7 (2C), 128.3, 126.4, 105.7, 96.1 (2C), 21.3; HRMS (EI) m/z calcd for $C_{16}H_{14}O_4$ [$M^{+\cdot}$] 270.0892 found 270.0895.

(*E*)-3-(*p*-tolyl)-1-(2,4,6-trihydroxyphenyl)prop-2-en-1-one **21** [5].
This chalcone was synthesized following the method C for phenol deprotection starting from **7** and was obtained with 84% yield, as an orange solid. Rf = 0.7 (PE/Acetone, 7:3); ^{1}H NMR (400 MHz, acetone-d_6) δ 12.01 (bs, 2H), 9.33 (bs, 1H), 8.22 (d, *J* = 15.7 Hz, 1H), 7.76 (d, *J* = 15.7 Hz, 1H), 7.58 (d, *J* = 7.7 Hz, 2H), 7.26 (d, *J* = 7.7 Hz, 2H), 5.98 (s, 2H), 2.36 (s, 3H); ^{13}C NMR (100 MHz, acetone-d_6) δ 193.2, 165.7 (2C), 165.6, 142.8, 141.3, 133.8, 130.5 (2C), 129.2 (2C), 127.5, 105.7, 96.1 (2C), 56.6.

(*E*)-3-(4-chlorophenyl)-1-(2,4,6-trihydroxyphenyl)prop-2-en-1-one **22** [5].
This chalcone was synthesized following the method C for phenol deprotection starting from **8** and was obtained as an orange solid, with 91% yield. Rf = 0.5 (DCM/MeOH, 95:5); ^{1}H NMR (400 MHz, acetone-d_6) 11.10 (bs, 3H), 8.24 (dd, *J* = 15.7 Hz, 3.4, 1H), 7.73 (d, *J* = 15.7 Hz, 1H), 7.70 (d, *J* = 8.6 Hz, 2H), 7.47 (d, *J* = 8.6 Hz, 2H), 5.98 (s, 2H); ^{13}C NMR (100 MHz, acetone-d_6) δ 192.9, 165.9, 165.8 (2C), 141.0, 136.1, 135.4, 130.6 (2C), 129.9 (2C), 129.3, 105.7, 96.1 (2C).

(*E*)-3-(2,4-dichlorophenyl)-1-(2,4,6-trihydroxyphenyl)prop-2-en-1-one **23** [5].
This chalcone was synthesized following the method C for phenol deprotection starting from **9** and was obtained as an orange solid, with 95% yield. Rf = 0.4

(hept/acetone, 3:2); ^{1}H NMR (400 MHz, acetone-d_6) δ 12.00 (bs, 2H), 9.56 (bs, 1H), 8.25 (d, J = 15.7 Hz, 1H), 8.03 (d, J = 15.7 Hz, 1H), 7.86 (d, J = 8.5 Hz, 1H), 7.61 (d, J = 2.1 Hz, 1H), 7.45 (dd, J = 8.5, 2.1 Hz, 1H), 5.98 (s, 2H); ^{13}C NMR (100 MHz, acetone-d_6) δ 192.6, 166.2, 165.8 (2C), 136.4, 136.2, 136.1, 133.5, 131.9, 130.5, 129.9, 128.8, 105.7, 96.1 (2C).

(*E*)-1-(2,4-dihydroxy-6-methoxyphenyl)-3-(4-hydroxyphenyl)prop-2-en-1-one **24** [6].
This chalcone was synthesized following the method D for phenol deprotection starting from **10** and was obtained as a yellow solid, with 55% yield. Rf = 0.4 (DCM/MeOH, 95:5); ^{1}H NMR (400 MHz, acetone-d_6) 14.33 (s, 1H), 7.85 (d, J = 15.5 Hz, 1H), 7.71 (d, J = 15.5 Hz, 1H), 7.58 (d, J = 8.5 Hz, 2H), 6.89 (d, J = 8.6 Hz, 2H), 6.04 (d, J = 2.3 Hz, 1H), 5.97 (d, J = 2.3 Hz, 1H), 3.94 (s, 3H); ^{13}C NMR (100 MHz, acetone-d_6) δ 192.3, 168.2, 164.8, 163.5, 159.8, 142.6, 130.5 (2C), 127.2, 124.4, 116.0 (2C), 105.5, 96.2, 91.3, 55.6.

(*E*)-3-(2,4-dichlorophenyl)-1-(2,4-dihydroxy-6-methoxyphenyl)prop-2-en-1-one **25**.
This chalcone was synthesized following the method D for phenol deprotection starting from **11** and was obtained as a yellow solid, with 23% yield. Rf = 0.5 (DCM/MeOH, 95:5); IR (ν cm^{-1}): 3350, 1633, 1594, 1464, 1341, 1201, 1112, 1100, 817; ^{1}H NMR (400 MHz, acetone-d_6) δ 13.97 (s, 1H), 9.66, (bs, 1H), 8.02 (s, 2H), 7.94 (d, J = 8.5 Hz, 1H), 7.60 (d, J = 2.1 Hz, 1H), 7.45 (dd, J = 8.5, 2.1 Hz, 1H), 6.08 (d, J = 2.1 Hz, 1H), 6.01 (d, J = 2.2 Hz, 1H), 3.96 (s, 3H); ^{13}C NMR (100 MHz, acetone-d_6) δ 192.5, 169.0, 166.5, 164.4, 136.4, 136.1(2C), 133.3, 131.9, 130.5, 130.1, 128.8, 106.3 97.0, 92.3, 56.5; HRMS (ESI) m/z calcd for $C_{16}H_{11}Cl_2O_4$ [M-H]$^-$ 337.0034 found 337.0036.

(*E*)-3-(4-fluorophenyl)-1-(2,4,6-trihydroxyphenyl)prop-2-en-1-one **26** [5].
This product was synthesized following the method D for phenol deprotection starting from **12** and was obtained as an orange solid, with 74% yield. Rf = 0.2 (PET/acetone, 8:2); ^{1}H NMR (400 MHz, acetone-d_6) 11.94 (s, 1H), 9.30 (s, 1H), 8.16 (d, J = 15.7 Hz, 1H), 7.76 – 7.68 (m, 3H), 7.17 (t, J = 8.8 Hz, 2H), 5.96 (s, 2H); ^{13}C NMR (100 MHz, acetone-d_6) δ 192.2, 164.9 (3C), 163.8 (d, $^1J_{C\text{-}F}$ = 249 Hz), 140.5, 132.3 (d, $^4J_{C\text{-}F}$ = 3,6 Hz), 130.5 (d, $^3J_{C\text{-}F}$ = 8,7 Hz, 2C), 127.6 (d, $^5J_{C\text{-}F}$ = 2.7 Hz), 115.9 (d, $^2J_{C\text{-}F}$ = 21.9 Hz, 2C), 104.9, 95.3 (2C); ^{19}F NMR (376 MHz, acetone-d_6) δ -111.8.

(*E*)-1-(2,4-dihydroxy-6-methoxyphenyl)-3-(4-fluorophenyl)prop-2-en-1-one **27** [7].
This chalcone was synthesized following the method D for phenol deprotection starting from **13** and was obtained as a yellow solid, with 79% yield. Rf = 0.2 (EDP/acetone, 9:1); ^{1}H NMR (400 MHz, acetone-d_6) 14.14 (s, 1H), 9.53 (s, 1H), 7.92 (d, J = 15.6 Hz, 1H), 7.80 – 7.63 (m, 3H), 7.17 (t, J = 8.8 Hz, 2H), 6.04 (d, J = 2.2 Hz, 1H), 5.98 (d, J = 2.3 Hz, 1H), 3.93 (s, 3H); ^{13}C NMR (100 MHz, acetone-d_6) δ 192.2, 168.2, 165.3, 163.8 (d, $^1J_{C\text{-}F}$ = 251.5 Hz), 163.6, 140.5, 132.2 (d, $^4J_{C\text{-}F}$ = 3.3 Hz), 130.6

(d, $^3J_{C-F}$ = 8.6 Hz, 2C), 127.6 (d, $^5J_{C-F}$ = 2.2 Hz), 115.9 (d, $^2J_{C-F}$ = 22.0 Hz, 2C), 105.5, 96.2, 91.4, 55.6; ^{19}F NMR (376 MHz, $CDCl_3$) δ -111.7.

(*E*)-3-(4-chlorophenyl)-1-(2,4-dihydroxy-6-methoxyphenyl)prop-2-en-1-one **28** [4].
This chalcone was synthesized following the method D for phenol deprotection starting from **14** and was obtained as a yellow solid, with 82% yield. Rf = 0.3 (EDP/acetone, 7:3); ^{1}H NMR (400 MHz, acetone-d_6) 14.09 (s, 1H), 9.56 (s, 1H), 7.96 (d, *J* = 15.6 Hz, 1H), 7.68 (t, *J* = 12.5 Hz, 3H), 7.42 (d, *J* = 8.3 Hz, 2H), 6.04 (s, 1H), 5.98 (s, 1H), 3.93 (s, 3H); ^{13}C NMR (100 MHz, acetone-d_6) δ 192.1, 168.2, 165.3, 163.6, 140.2, 135.3, 134.5, 129.9 (2C), 129.1 (2C), 128.4, 105.5, 96.2, 91.5, 55.6.

(*E*)-3-(4-hydroxyphenyl)-1-(2,4,6-trihydroxyphenyl)prop-2-en-1-one **29** [9].
This product was synthesized following the method D for phenol deprotection starting from **15** and was obtained as a yellow solid, with 49% yield. Rf = 0.2 (DCM/MeOH, 98:2); ^{1}H NMR (400 MHz, acetone-d_6) 12.02 (s, 1H), 9.25 (s, 1H), 8.91 (s, 1H), 8.10 (d, *J* = 15.6 Hz, 1H), 7.74 (d, *J* = 15.5 Hz, 1H), 7.54 (d, *J* = 5.7 Hz, 2H), 6.88 (d, *J* = 5.4 Hz, 2H), 5.94 (s, 2H); ^{13}C NMR (100 MHz, acetone-d_6) δ 192.4, 164.9 (2C), 164.6, 159.7, 142.5, 130.4 (2C), 127.3, 124.4, 116.0 (2C), 104.9, 95.2 (2C).

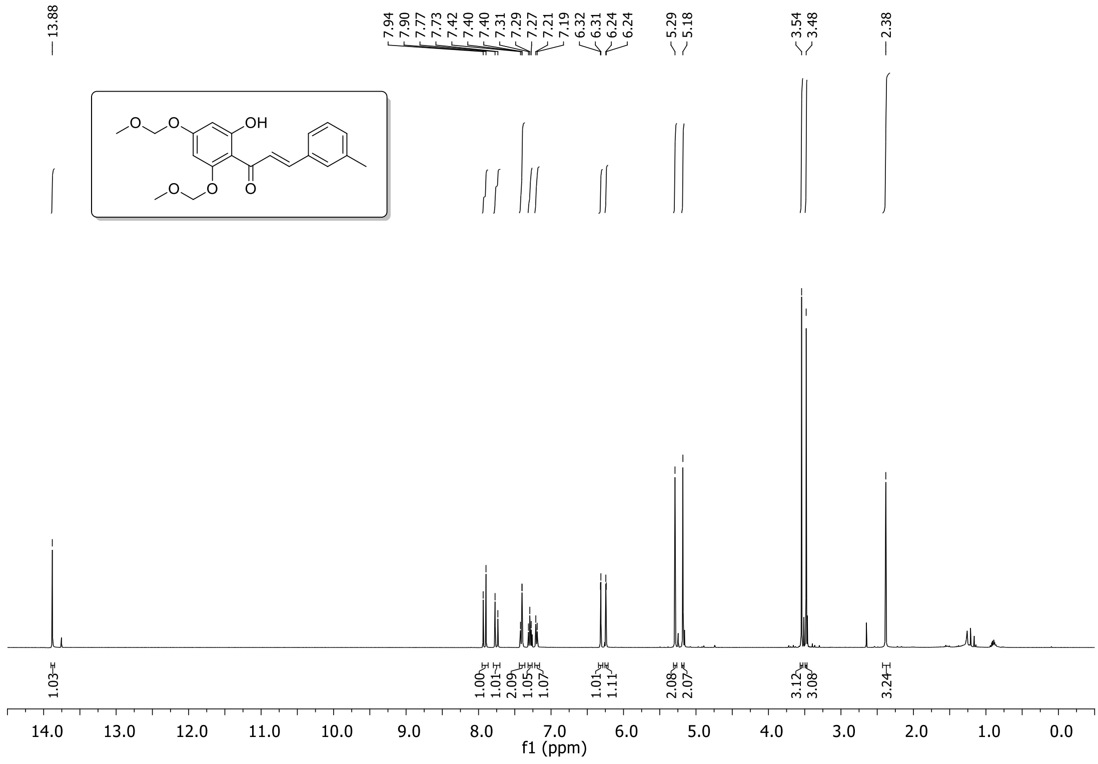

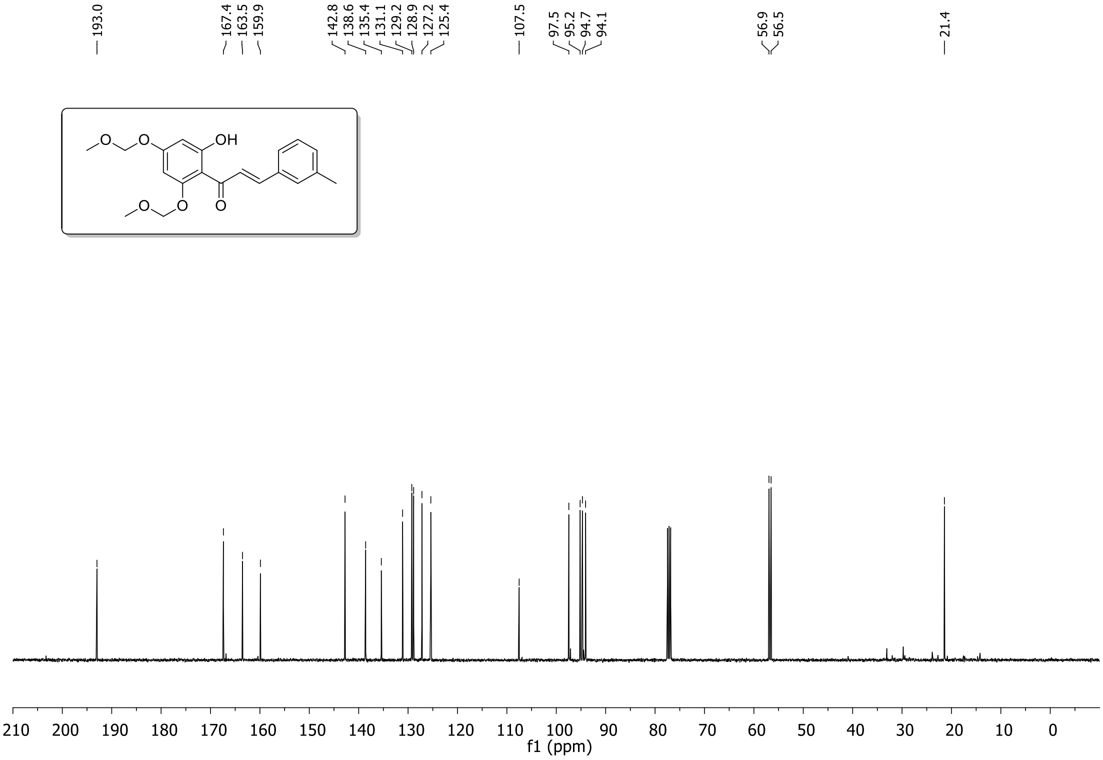

Figure S2: ^{1}H and ^{13}C NMR spectra of **6** in $CDCl_3$

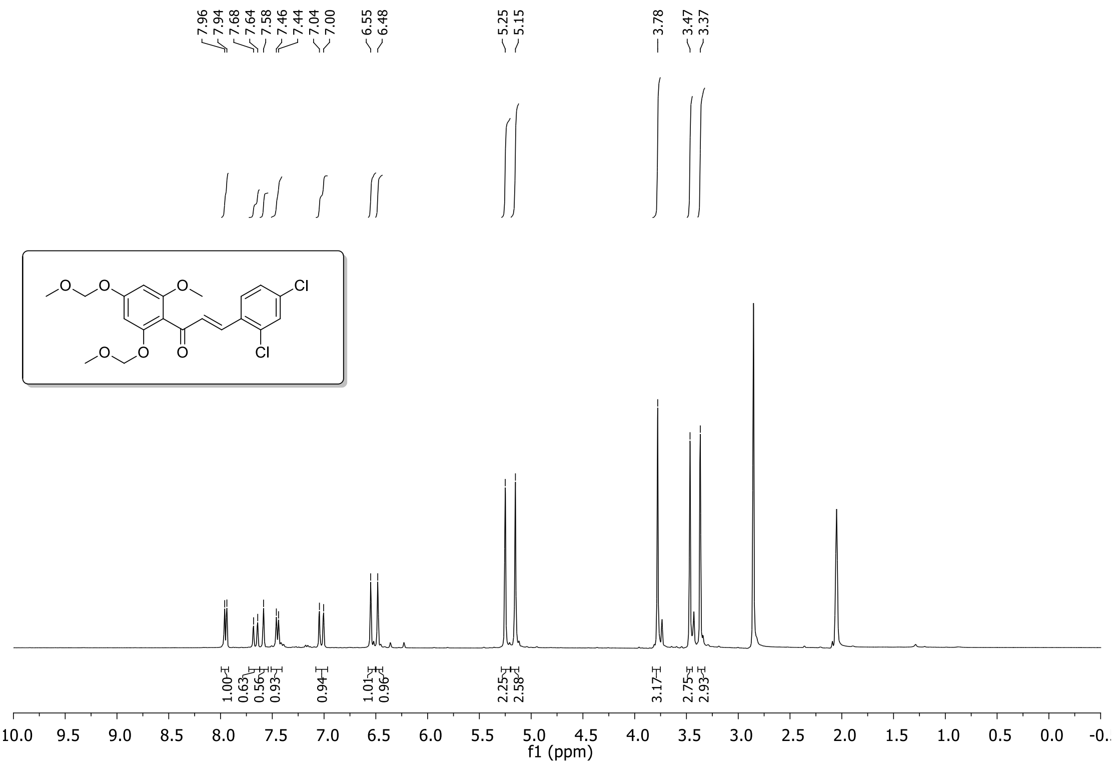

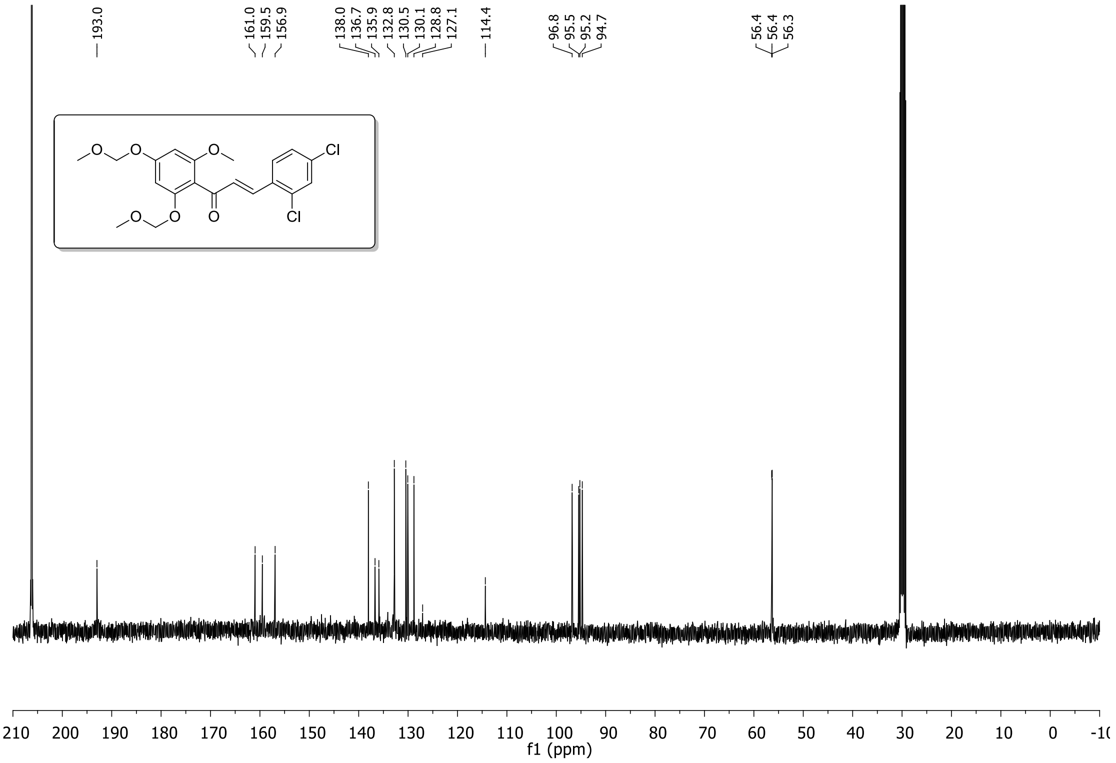

Figure S3: ^{1}H and ^{13}C NMR spectra of **11** in acetone-d_6

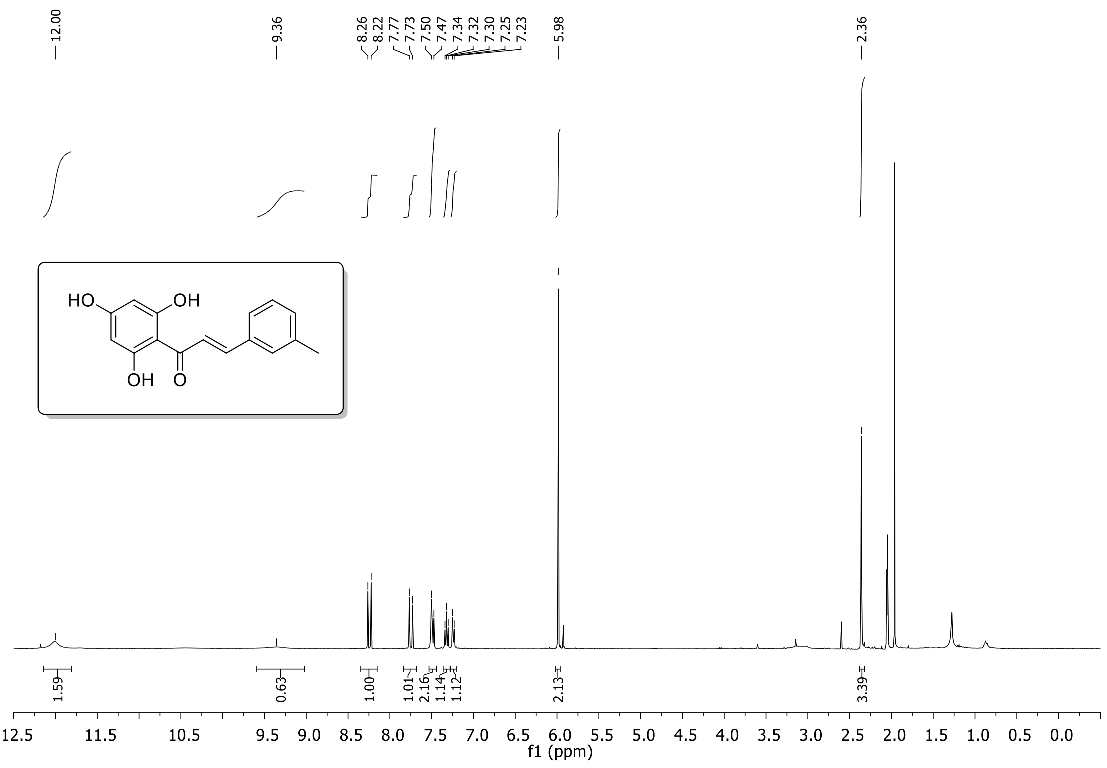

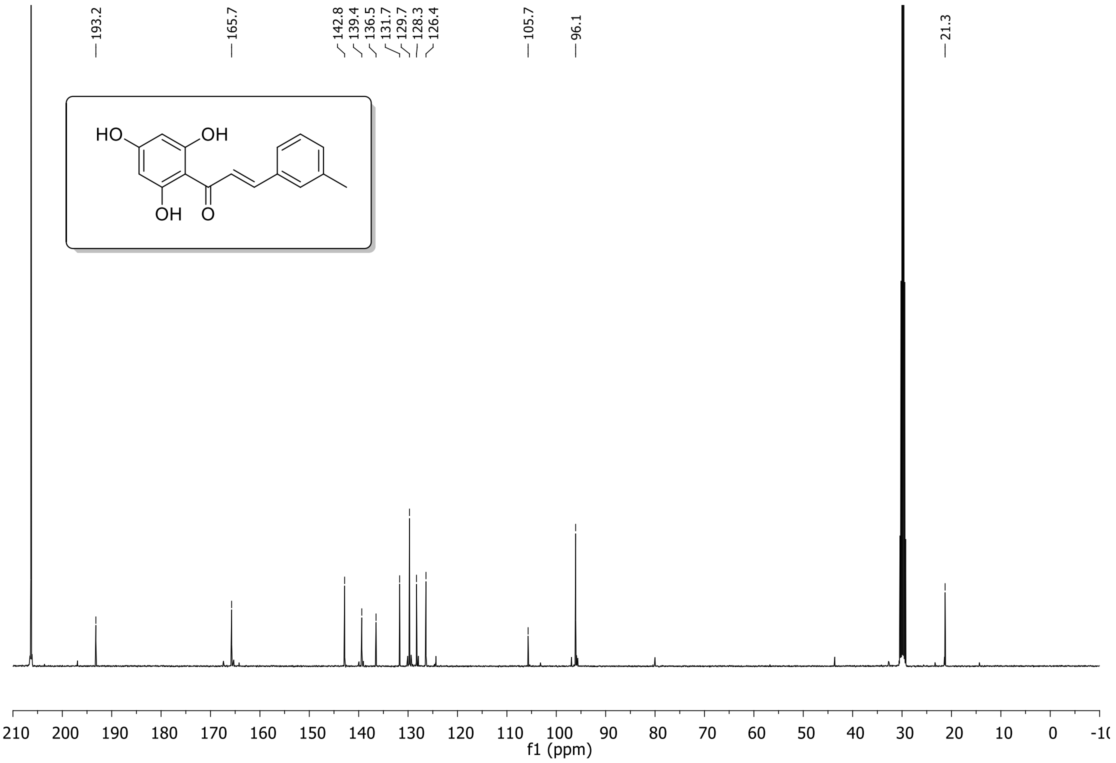

Figure S4: ^{1}H and ^{13}C NMR spectra of **20** in acetone-d_6

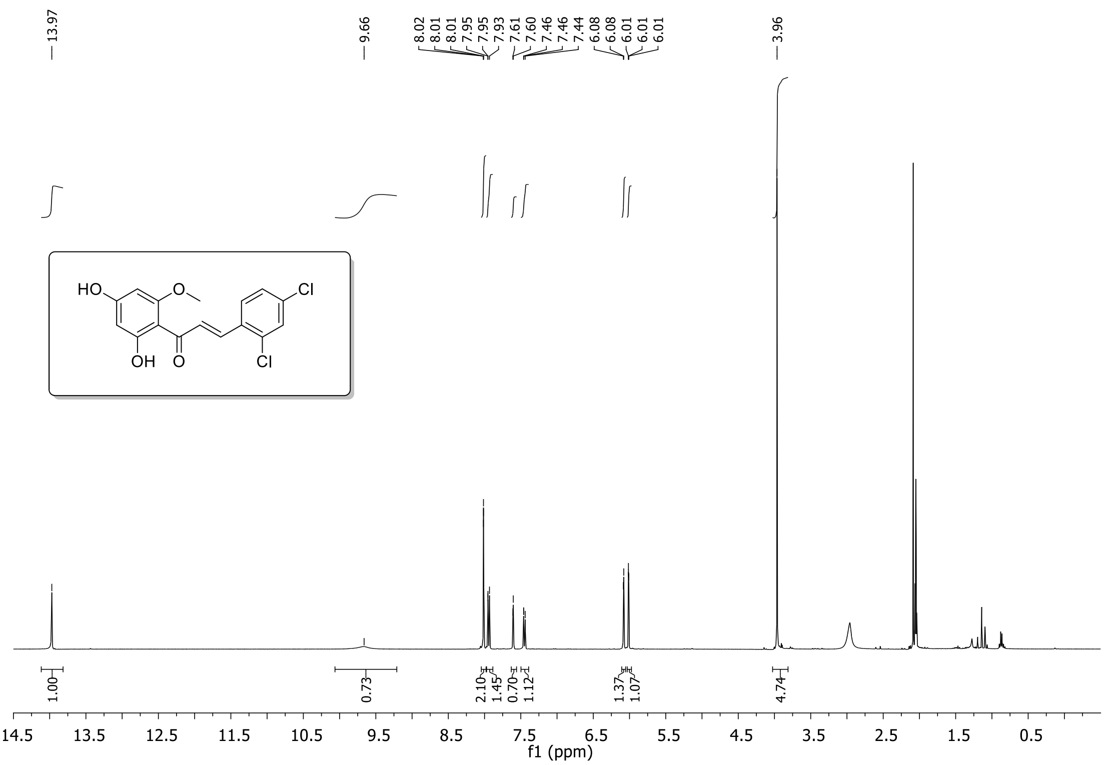

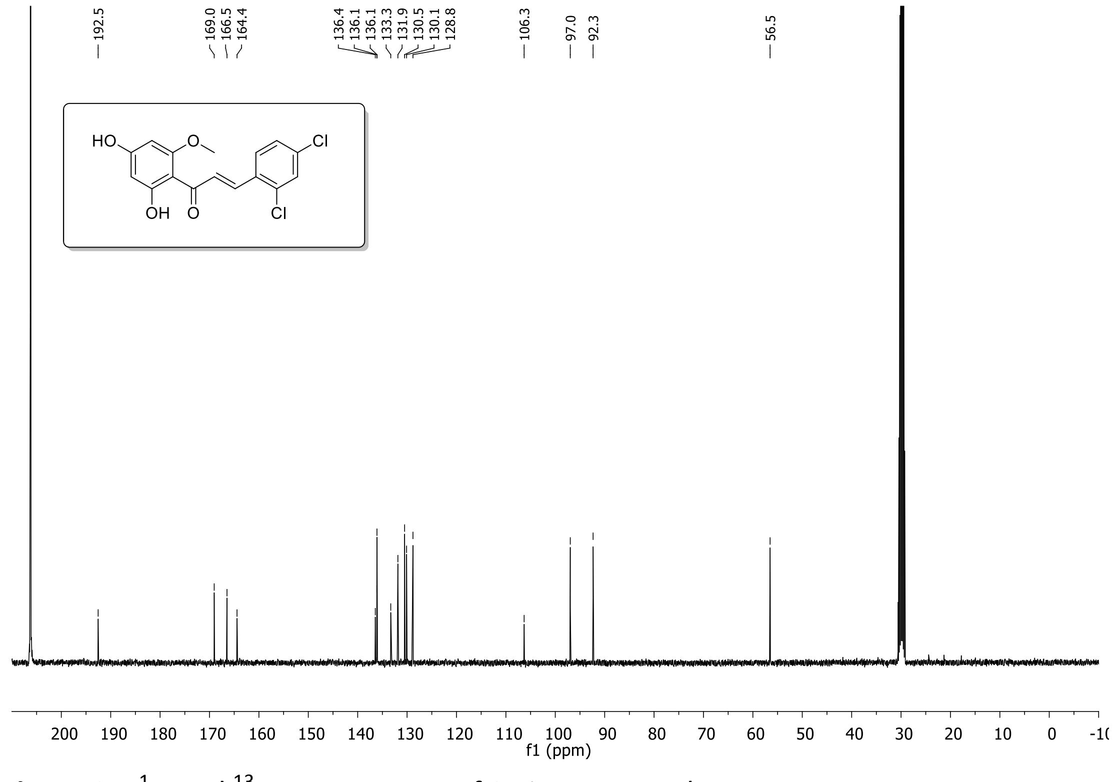

Figure S5: ^{1}H and ^{13}C NMR spectra of **25** in acetone-d_6

Table S2: Chalcone structures and inhibition data.

Compound	Structure	AKR1C3 Inhibition at 10 µM [%]	AKR1C3 Inhibition - IC_{50} [µM]
isoliquiritigenin (**16**)	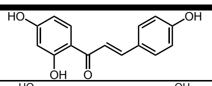	31.1 ± 3.2	nd
butein (**17**)	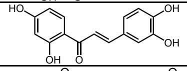	37.2 ± 1.4	nd
**18** (MF-11)	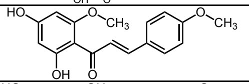	**47.3 ± 6.4**	**11.91 ± 2.03**
**19**	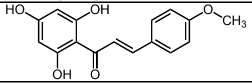	**78.1 ± 2.7**	**2.36 ± 0.54**
**20**	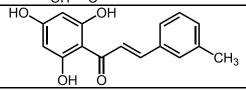	**89.2 ± 6.3**	**1.94 ± 0.32**
**21**	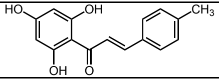	**87.9 ± 4.9**	**5.18 ± 1.64**
**22**	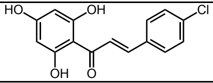	70.8 ± 3.8	nd
**23**	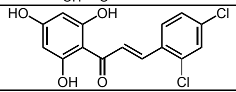	**90.9 ± 4.6**	**1.08 ± 0.27**
**24**	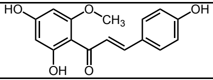	24.6 ± 4.8	nd
**25**	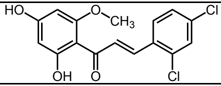	**33.3 ± 6.0**	**nd**
**26**	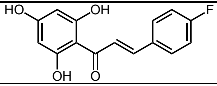	68.5 ± 6.4	nd
**27**	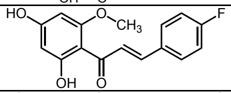	20.3 ± 8.4	nd
**28**	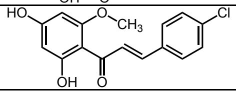	26.1 ± 2.0	nd
**29**	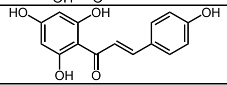	40.6 ± 7.7	nd

Data for chalcones analyzed in more detail are written in bold; nd = not determined

Table S3: Compound selectivity - Inhibition of human AKR1C enzymes by chalcones at 10 µM concentration in %

Compound	AKR1C3	AKR1C1	AKR1C2	AKR1C4
**18** (MF11)	47.3 ± 6.4	96.5 ± 0.9	21.4 ± 5.1	25.0 ± 1.5
**19**	78.1 ± 2.7	96.3 ± 1,2	38.5 ± 1.3	93.3 ± 0.7
**20**	89.2 ± 6.3	86.9 ± 1.1	39.9 ± 4.0	79.6 ± 0.2
**21**	87.9 ± 4.9	90.2 ± 0.8	42.3 ± 2.1	91.6 ± 1.5
**23**	90.9 ± 4.6	97.0 ± 0.5	94.0 ± 0.4	97.0 ± 0.9
**25**	33.3 ± 6.0	87.9 ± 0.6	38.5 ± 3.9	41.6 ± 1.8

Table S4: Compound selectivity - Inhibition of human SDR enzymes primarily involved in steroid metabolism by chalcones at 10 µM concentration in %

Compound	HSD17B1	HSD17B2	HSD17B3
**18** (MF11)	46.5 ± 8.8	10.4 ± 1.6	24.8 ± 4.8
**19**	42.3 ± 5.3	29.6 ± 3.7	41.8 ± 3.3
**20**	51.8 ± 5.1	61.0 ± 0.9	55.9 ± 4.1
**21**	42.8 ± 9.1	44.2 ± 4.7	52.7 ± 6.7
**23**	53.3 ± 8.8	88.8 ± 0.2	68.6 ± 4.7
**25**	-1.9 ± 17.1	12.7 ± 2.3	20.9 ± 8.0

Table S5: Compound selectivity - Inhibition of human SDR enzymes primarily involved in other pathways than steroid metabolism by chalcones at 10 µM concentration in %

Compound	HSD17B4	HSD17B7	HSD17B10	HSD17B14
**18** (MF11)	9.9 ± 6.1	6.5 ± 6.7	-0.5 ± 6.5	24.9 ± 8.1
**19**	2.0 ± 2.9	4.6 ± 4.4	2.2 ± 2.6	1.8 ± 7.7
**20**	8.6 ± 1.1	3.8 ± 3.5	-2.5 ± 0.7	18.5 ± 9.3
**21**	3.0 ± 6.3	6.8 ± 4.7	8.0 ± 6.5	-8.0 ± 2.3
**23**	19.6 ± 1.0	9.9 ± 4.7	5.2 ± 3.8	13.9 ± 2.7
**25**	-6.0 ± 7.5	37.7 ± 11.4	-0.5 ± 7.3	-3.2 ± 6.3

Table S6: Activity assay setup

Human enzyme expression construct	Assayed reaction	Radioactive substrate; concentration in assay	Co-factor concentration in assay	Assay buffer
**reductive reactions**				
pGEX HSD17B1 in *E. coli*	E1 → E2	estrone-[6,7-^{3}H]; 20 nM	NADPH 600µM	NaPi pH6.6
pGEX HSD17B7 in *E. coli*	E1 → E2	estrone-[6,7-^{3}H]; 40 nM	NADPH 600µM	NaPi pH7.7, 1mM EDTA, 0.05% BSA
HSD17B3 stable in HEK293	A → T	Δ4-androstenedione - [1,2,6,7-^{3}H]; 11.9 nM	NADPH 600µM	NaPi pH6.6 + PI/1mM EDTA
pGEX-AKR1C3 in *E.coli; Lysate*	A → T	Δ4-androstenedione - [1,2,6,7-^{3}H]; 11.9 nM	NADPH 600µM	NaPi pH6.6
pDEST-Nmyc-AKR1C1 in HEK293	P → 20αOHP	progesterone-[1,2-^{3}H]; 40 nM	NADPH 600µM	NaPi pH7.4 + PI/1mM EDTA
pDEST-Nmyc-AKR1C2 in HEK293	DHT → 3α-Adiol	dihydrotestosterone-[1,2-^{3}H]; 16.7 nM	NADPH 600µM	NaPi pH7.4 + PI/1mM EDTA
pDEST-N-myc-AKR1C4 in HEK 293	DHT → 3α-Adiol	dihydrotestosterone-[1,2-^{3}H]; 16.7 nM	NADPH 600µM	NaPi pH7.4 + PI/1mM EDTA
**oxidative reactions**				
pGEX-HSD17B2 in *E.coli*	E2 → E1	estradiol-[6,7-^{3}H], 40 nM	NAD 750 µM	NaPi pH7.7
pGEX-HSD17B4 (SDR domain) in *E.coli*	E2 → E1	estradiol-[6,7-^{3}H], 40 nM	NAD 750 µM	NaPi pH7.7
pGEX-HSD17B10 in *E.coli*	E2 → E1	estradiol-[6,7-^{3}H], 40 nM	NAD 750 µM	NaPi pH7.7
p11-HSD17B14 (S205) in *E.coli*	E2 → E1	estradiol-[6,7-^{3}H], 40 nM	NAD 750 µM	NaPi pH8.0

E1: estrone; E2: 17β-estradiol, A: Δ4-androstene-3,17-dione; T: testosterone, P: progesterone, 20αOHP: 20α-hydroxyprogesterone, DHT: dihydrotestosterone, 3α-Adiol: 3α-androstanediol, NaPi: 100 mM Na-phosphate buffer; PI: C0mplete protease inhibitor (Roche);. *E.coli* - Expression in *E.coli* BL21 DE3 Codon Plus RP except *E.coli* BL21 DE3 for HSD17B14.

References:

1. Urgaonkar, S.; La Pierre, H.S.; Meir, I.; Lund, H.; RayChaudhuri, D.; Shaw, J.T. Synthesis of antimicrobial natural products targeting FtsZ: (+/-)-Dichamanetin and (+/-)-2'''-hydroxy-5''-benzylisouvarinol-B. *Org Lett* **2005**, *7*, 5609-5612, doi:10.1021/ol052269z.
2. Thevenin, M.; Mouray, E.; Grellier, P.; Dubois, J. Facile formation of methylenebis(chalcone)s through unprecedented methylenation reaction. application to antiparasitic and natural product synthesis. *Eur J Org Chem* **2014**, *2014*, 2986-2992, doi:10.1002/ejoc.201400104.
3. Flaherty, D.P.; Kiyota, T.; Dong, Y.X.; Ikezu, T.; Vennerstrom, J.L. Phenolic bis-styrylbenzenes as beta-amyloid binding ligands and free radical scavengers. *J Med Chem* **2010**, *53*, 7992-7999, doi:10.1021/jm1006929.
4. Zhang, B.X.; Duan, D.Z.; Ge, C.P.; Yao, J.; Liu, Y.P.; Li, X.M.; Fang, J.G. Synthesis of Xanthohumol analogues and discovery of potent thioredoxin reductase inhibitor as potential anticancer agent. *J Med Chem* **2015**, *58*, 1795-1805, doi:10.1021/jm5016507.

5. Sui, X.; Quan, Y.C.; Chang, Y.; Zhang, R.P.; Xu, Y.F.; Guan, L.P. Synthesis and studies on antidepressant activity of 2',4',6'-trihydroxychalcone derivatives. *Med Chem Res* **2012**, *21*, 1290-1296, doi:10.1007/s00044-011-9640-2.
6. Vogel, S.; Ohmayer, S.; Brunner, G.; Heilmann, J. Natural and non-natural prenylated chalcones: Synthesis, cytotoxicity and anti-oxidative activity. *Bioorg Med Chem* **2008**, *16*, 4286-4293, doi:10.1016/j.bmc.2008.02.079.
7. Jin, Y.L.; Jin, X.Y.; Jin, F.; Sohn, D.H.; Kim, H.S. Structure activity relationship studies of anti-inflammatory TMMC derivatives: 4-dimethylamino group on the B ring responsible for lowering the potency. *Arch Pharm Res* **2008**, *31*, 1145-1152, doi:10.1007/s12272-001-1281-7.
8. Nguyen, V.S.; Dong, L.P.; Wang, S.C.; Wang, Q.A. The first total synthesis of Sophoflavescenol, Flavenochromane C, and Citrusinol. *Eur J Org Chem* **2015**, *2015*, 2297-2302, doi:10.1002/ejoc.201403689.
9. Jeong, S.; Lee, S.; Kim, K.; Lee, Y.; Lee, J.; Oh, S.; Choi, J.W.; Kim, S.W.; Hwang, K.C.; Lim, S. Isoliquiritigenin derivatives inhibit RANKL-Induced osteoclastogenesis by regulating p38 and NF-kappa B activation in RAW 264.7 cells. *Molecules* **2020**, *25*, 3908 doi:10.3390/molecules25173908.
